# Application of visible light active photocatalysis for water contaminants: A review

**DOI:** 10.1002/wer.10781

**Published:** 2022-10-04

**Authors:** Yifan Sun, David W. O'Connell

**Affiliations:** ^1^ Department of Civil and Environmental Engineering Trinity College Dublin Dublin 2 Ireland

**Keywords:** organic pollutants, pilot‐scale applications, visible light active photocatalyst, water treatment

## Abstract

Organic water pollutants are ubiquitous in the natural environment arising from domestic products as well as current and legacy industrial processes. Many of these organic water pollutants are recalcitrant and only partially degraded using conventional water and wastewater treatment processes. In recent decades, visible light active photocatalyst has gained attention as a non‐conventional alternative for the removal of organic pollutants during water treatment, including industrial wastewater and drinking water treatment. This paper reviews the current state of research on the use of visible light active photocatalysts, their modified methods, efficacy, and pilot‐scale applications for the degradation of organic pollutants in water supplies and waste streams. Initially, the general mechanism of the visible light active photocatalyst is evaluated, followed by an overview of the major synthesis techniques. Because few of these photocatalysts are commercialized, particular attention was given to summarizing the different types of visible light active photocatalysts developed to the pilot‐scale stage for practical application and commercialization. The organic pollutant degradation ability of these visible light active photocatalysts was found to be considerable and in many cases comparable with existing and commercially available advanced oxidation processes. Finally, this review concludes with a summary of current achievements and challenges as well as possible directions for further research.

**Practitioner Points:**

- Visible light active photocatalysis is a promising advanced oxidation process (AOP) for the reduction of organic water pollutants.
- Various mechanisms of photocatalysis using visible light active materials are identified and discussed.
- Many recent photocatalysts are synthesized from renewable materials that are more sustainable for applications in the 21st century.
- Only a small number of pilot‐scale applications exist and these are outlined in this review.

## Graphical abstract

Visible light active photocatalysis has gained attention as an effective non‐conventional alternative technique for the degradation of organic pollutants during water treatment, including industrial wastewater and drinking water treatment. Currently, there is a need for greater adoption and upscaling of these techniques to the pilot scale, which can often be more effective and energy efficient than conventional techniques.

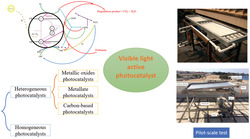

## INTRODUCTION

Water pollution has serious negative impacts on both aquatic environments and associated human activities. The effluent from industry, agriculture, hospital, and households may contain many organic pollutants, such as dyes, paints, pesticides, gasoline, and pathogen (Cunha et al., 2019; Priyanka et al., 2020; Tran et al., 2014; Vatanpour et al., 2019). Some of them are recalcitrant and can only partially be treated through the traditional wastewater treatment process; examples are shown in Table 1. Due to rapid population increase, global industrialization, and urbanization, clean water resources are under pressure due to large increases in demand over the past number of decades. Organic pollutants discharged from such activities and sources have exacerbated the problem and many traditional wastewater treatment methods have been studied and applied to tackle this, including biotreatment, chemical treatment, filtration, and adsorption. However, such traditional wastewater treatment methods have limitations and disadvantages in treating water laden with persistent organic pollutants (Pardeshi & Patil, 2008; Villaluz et al., 2019); hence, advanced oxidation processes (AOPs) have been developed to improve treatment performance.

**TABLE 1:** Examples of recalcitrant pollutants

Categories	Pollutants	Reference
Textile	Axo dyes	(Raman & Kanmani, 2019)
	Alizarin yellow R (AYR)	(Ahmed et al., 2020)
	Laccases	(Giovanella et al., 2020)
Pharmaceutical	Levofloxacin (LEV)	(Wang, Chen, et al., 2020)
	Sulfamethoxazole	(Domingues et al., 2020)
	Cotinine	(Chavez et al., 2020)
	Benzodiazepines	(Cunha et al., 2019)
	Naproxen (NPX)	(Amini et al., 2019)
	Acetaminophen (ACE)	(Tobajas et al., 2017)
Industrial	Coffee processing wastewater	(Sujatha et al., 2020)
	Trichloroethylene (TCE)	(Jung et al., 2020)
	Phenol	(Scott et al., 2019)
	Chloro‐phenol	(Villaluz et al., 2019)
	Bisphenol A (BPA)	(Lim et al., 2019)
Pesticides	Malathion	(Vela et al., 2018b)
	Acetamiprid	(Garrido et al., 2020)

AOPs rely on the in situ generation of high oxidant species such as hydroxyl radicals that completely mineralize organic pollutants. This has been recognized as a promising water treatment solution, especially for those refractory contaminants (e.g., phenolic compounds, adsorbable organic halides, bisphenol A [BPA], and antibiotics) (Cai et al., 2019; Garcia‐Segura et al., 2018; Oh et al., 2016; Villaluz et al., 2019; Zhang et al., 2020). In the past several decades, many AOPs have been developed, such as peroxide, electrochemical, Fenton, sonolysis, microwave, and photocatalytic (Miklos et al., 2018; Zhang et al., 2020) (Table 2).

**TABLE 2:** AOP technology and associated advantages and disadvantages

AOP technology	Advantages	Disadvantages	Reference
Electrochemical oxidation	No chemical compounds created Versatility Scalability	Consumes electricity	(Krzeminska et al., 2015) (Garcia‐Segura et al., 2018)
Sonolysis	Low interference from the water matrix Less heat transfer	Highly energy intensive Low electrical efficiency	(Pang et al., 2011)
Microwave	Enhance reaction rates reduce selective heating	Low electrical efficiency Required cooling devices	(Pang et al., 2011) (Miklos et al., 2018)
Fenton	The high contaminants removal efficiency	Chemical consumption	(Babuponnusami & Muthukumar, 2012) (Krzeminska et al., 2015) (Bokare & Choi, 2014)
Fenton‐like oxidation process	Low cost	Each non‐ferrous catalyst has its merits and demerits Limited application	(Bokare & Choi, 2014) (Garrido‐Ramirez et al., 2010)
Ozonization	High degradation and mineralization efficiency	Not effective for recalcitrant organics	(Malik et al., 2020)

Among all AOPs, photocatalysis has received increasing attention, due to its lower cost, non‐toxic materials, relatively high chemical stability of the catalyst, and efficiency under mild conditions using potential sunlight (Miklos et al., 2018; Xu et al., 2020; Yusuff et al., 2020). In addition, it also has a high potential for complete degradation, destruction, or mineralization of organic pollutants (dos Santos et al., 2019; Gmurek et al., 2019; Katal et al., 2019).

Photocatalysis may be divided into two categories, ultraviolet (UV) active photocatalysis and solar/visible light active photocatalysis, which has become a preferred choice (Shaniba et al., 2020; Sujatha et al., 2020). Although UV photocatalysis generally has better treatment performance, as the intensity of UV is stronger than solar/visible light's (Sujatha et al., 2020), but due to the economics of solar/visible light photocatalysis, it is preferred over UV light photocatalysis and has been considered an environmentally friendly technology for pollutant removal.

Recently, significant progress has been made on the visible light active photocatalysts. This review summarizes the latest developments in visible light active photocatalysis. It starts with the mechanism of photocatalysis, followed by synthesis and doping methods of several common photocatalysts, and the application of visible light active photocatalysis. Special attention has been devoted to pilot‐scale tests and introduced separately. Finally, current research deficiencies and prospects for future research are considered. We believe that this review will not only promote the further developments of visible light active photocatalysis but also could therefore help to address the attention for the pilot‐scale tests and even real‐world application of visible light active photocatalysts in water treatment.

## VISIBLE LIGHT ACTIVE PHOTOCATALYSTS

Visible light active photocatalysis is a type of AOP based on the generation of radicals after photoexcitation of a semiconductor material (Zuniga‐Benitez & Penuela, 2020). In general, the visible light active photocatalyst consists of semiconductor materials, light‐harvesting antennas, and active species (Dong et al., 2015; Waso et al., 2020; Xu et al., 2020). The mechanism and materials are critical information for photocatalysis research. In this section, the mechanism of photocatalytic function and materials have been reviewed.

### Mechanism of photocatalytic function

In general, the mechanism of visible light active photocatalysis is using solar/visible energy to create radical and other active species and then degrade pollutants. The series of actions possibly happened at the visible light active photocatalysts due to light absorption for pollutant degradation, which has been intensively reported in much literature (Ali et al., 2019; Bibova et al., 2019; Chong et al., 2010; Dong et al., 2015; Fujishima et al., 2008; Pena et al., 2005) and it summarized as follows:

(1) $$\text{Photocatalyst}+\mathrm{h\nu}\to \text{Photocatalyst}\;\left(h^{+}+e^{-}\right)$$

(2) $$h^{+}+H_{2}O\to \cdot \mathrm{OH}+H^{+}$$

(3) $$h^{+}+{\mathrm{OH}}^{-}\to \cdot \mathrm{OH}$$

(4) $$e^{-}+O_{2}\to \cdot {O_{2}}^{-}$$

(5) $${O_{2}}^{-}+H^{+}\to \cdot \mathrm{OOH}$$

(6) $$\cdot \mathrm{OOH}+\cdot {O_{2}}^{-}\to {\mathrm{OOH}}^{-}+O_{2}$$

(7) $${\mathrm{OOH}}^{-}+h^{+}\to \cdot \mathrm{OOH}$$

(8) $$2\cdot \mathrm{OOH}\to O_{2}+H_{2}O_{2}$$

(9) $$H_{2}O_{2}+\cdot {O_{2}}^{-}\to \cdot \mathrm{OH}+{\mathrm{OH}}^{-}+O_{2}$$

(10) $$H_{2}O_{2}+\mathrm{h\nu}\to 2\cdot \mathrm{OH}$$

(11) $$\text{Pollutant}+\left(\cdot \mathrm{OH},h^{+},\cdot \mathrm{OOH},\text{or}\;{O_{2}}^{-}\right)\to \text{degradation product}+{\mathrm{CO}}_{2}+H_{2}O$$

(12) $$h^{+}+e^{-}\to \text{heat}$$

**FIGURE 1:**
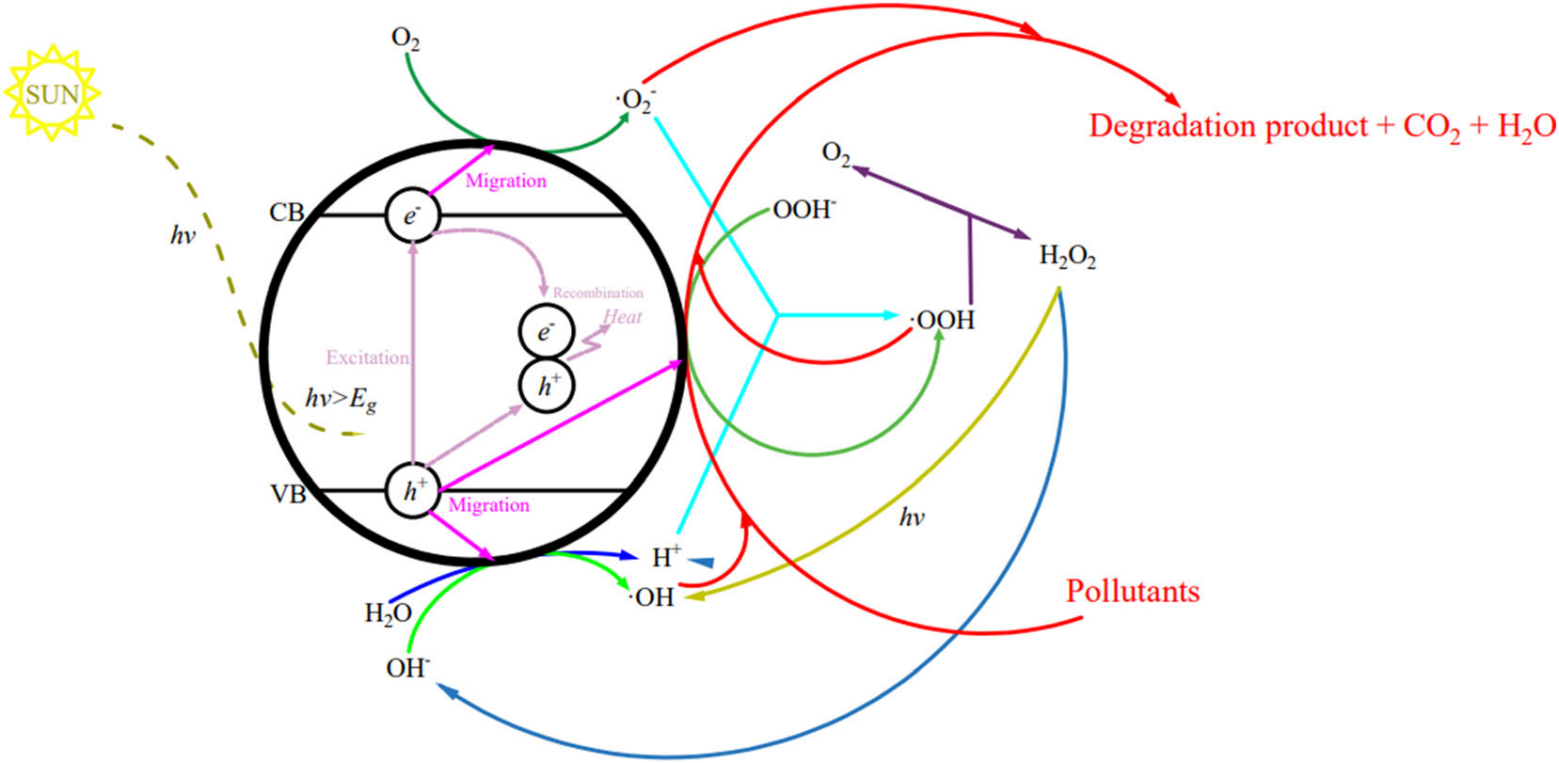
Photocatalytic reaction mechanism

If visible light energy (*hν*, *ν* is light's frequency, *h* is called Planck's constant and equal to 6.62608 × 10^−34^ Js) absorbed by the photocatalyst is stronger than its band gap energy (E_g_), valence band (VB) electrons (e^−^) will be excited to the conduction band (CB) and leave behind photogenerated holes (h^+^) at the VB (Equation 1) (Dong et al., 2015; Jung et al., 2020). Then, the produced e^−^/h^+^ will migrate to the surface and participate in a range of redox reactions shown above (Fujishima et al., 2008; Perović et al., 2020). Dong et al. (2015) summarized the three main active species for photocatalytic: h^+^, hydroxyl radical (·OH), and superoxide radical (·O_2_^−^), where ·OH has been considered the primary oxidant with scavenging properties (Chong et al., 2010). ·OH is generated by three routes: (1) H_2_O oxidized by h^+^ form H^+^ and ·OH (Equation 2); (2) O_2_ in the aqueous solution is reduced by e^−^ to form ·O_2_^−^ (Equation 4), which reacts with H^+^ and form ·OOH (Equation 5). ·OOH decomposes into O_2_ and H_2_O_2_ (Equation 8), which is continually reduced by ·O_2_^−^ to OH^−^ (Equation 9) and forms ·OH by h^+^ (Equation 3); and (3) H_2_O_2_ decomposed by light forming ·OH directly (Equation 10). Pollutants degraded by ·OH, h^+^, and ·O_2_^−^ produce degradation products, CO_2_ and H_2_O. If the of h^+^ with e^−^ and h^+^ are not quickly cleared after photoexcitation, it will lead to recombination. The recombination of h^+^ with e^−^ release heat/energy (Equation 11) has a negative impact on photocatalysis (Fujishima et al., 2008). The photocatalytic reaction mechanism is shown in Figure 1.

The mechanism of modified photocatalyst composites, except for the reactions above, usually includes the transfer of e^−^ and h^+^ between different photocatalysts, due to the different band potential (Geng et al., 2019; B. S. Li, Lai, et al., 2019). A good design of composite will lead to optimized parameters (like broadened energy band), efficient separation of photogenerated electron–hole pairs, and charge carrier recombination hindrance (Meena et al., 2020).

To capture solar energy effectively and achieve the above reactions, the semiconductor material for photocatalytic must have several critical properties. First, semiconductor materials should have a narrow band gap to absorb visible light effectively (Gopalakrishnan et al., 2020); the band gap determines the wavelength of light that materials can be absorbed (Casbeer et al., 2012). Secondly, the semiconductor materials should have a low recombination rate of h^+^–e^−^ pairs to ensure h^+^ and e^−^ migrate to the surface (Shaniba et al., 2020). Moreover, the semiconductor material should have good chemical and structural stability with the ability to operate under normal temperature and pressure (Chong et al., 2010). Other preferable properties include low cost, good stability, non‐toxicity, desired environmental acceptance, and easy to be separated (Xu et al., 2020). The choice of semiconductor material is crucial for photocatalysis.

Overall, the design of a good photocatalyst involves two key factors: (1) suitable semiconductor materials, preferable characteristics including good chemical and structural stability, solar energy absorption properties, and separation efficiency of charge carriers; and (2) favorable surface areas structure design of the photocatalyst (Berger et al., 2020); generally, the higher surface areas will lead to improved solar absorption and higher reaction rate but may leave traces as residue in water (Sharmila et al., 2020). Two common methods to fabricate better photocatalysts are modifying known materials and developing new semiconductor materials.

### Photocatalysis materials

Photocatalysts can be divided into two categories which include heterogeneous and homogeneous catalysts. Heterogeneous photocatalyst is in a different phase from the reactants (e.g., TiO_2_ for water treatment) (Ameta et al., 2018). On the contrary, a homogeneous photocatalyst is an assembly of soluble molecular catalysts in the same phase as the reactants, and all reactions are carried out in a single form (e.g., photo‐Fenton [PF] reagent for water treatment) (Shwetharani & Balakrishna, 2014; Tahir et al., 2020). Furthermore, heterogeneous catalysts may be divided into metallic compounds including carbon‐based compounds and metal‐carbon composites.

The metallic compound is very popular as a photocatalyst material. Generally, titanium dioxide (TiO_2_) and zinc oxide (ZnO) are the most common materials for photocatalysis, which have been widely used in the past few decades, due to their chemical stability, non‐toxic nature, abundance, and cost‐effectiveness (Priyanka et al., 2020; Shaniba et al., 2020; Xu et al., 2020; Yazdanbakhsh et al., 2019). Moreover, they can be easily prepared by simple and scalable approaches (Serra, Zhang, et al., 2019; Wang et al., 2014). However, TiO_2_ can only absorb UV light, which is only approximately 3%–5% of the entire light emitted across the solar spectrum (wavelengths shorter than 400 nm) because of its large band gap energy (3–3.2 eV) and fast charge recombination of h^+^ and e^−^ before reaching the surface. In addition, TiO_2_ powder is hard to separate from the reaction system. These defects lead to poor photocatalytic efficiency (Jung et al., 2020; Kavil et al., 2020; Shaniba et al., 2020; Weon et al., 2019; Xu et al., 2020). ZnO also has similar drawbacks; it has a wide band gap energy (3.3 eV), high photocorrosion activity, high electron mobility (200–300 cm^2^/V/s), low quantum efficiency, high h^+^ and e^−^ recombination rate, long electron lifetime (>10 s) (Ani et al., 2018; Hu et al., 2019; Serra, Gomez, & Philippe, 2019), and potential ecotoxicological effects (Serra et al., 2020). In addition, in this particular case, ZnO can cause ecotoxicological effects, especially relevant for some microorganisms, such as microalgae (Djearamane et al., 2018; Shahid et al., 2020).

Through hydrogenation of TiO_2_, it is possible to generate a black TiO_2_ solution. Black TiO_2_ has recently been developed, first reported in 2011 (X. Chen et al., 2011), and has triggered much research interest in recent years. It displays excellent red‐shifted light absorption properties, mainly owing to hydrogenation dramatically changing the structural, chemical, electronic, and optical properties of TiO_2_ nanoparticles (NPs), so it has a stronger visible light utilization ability (J. U. Choi et al., 2019; Y. Liu et al., 2017). However, according to the latest review, summarized by Rajaraman et al. (2020), the synthesis of black TiO_2_ is quite challenging and, in some cases, compared with pristine TiO_2_, the black TiO_2_ actually showed reduced photoactivity.

Another common solution is doping, coating, and decorating elements into TiO_2_ and ZnO (Scott et al., 2019; Shaniba et al., 2020; Villaluz et al., 2019). These elements can act as a sink for h^+^ and e^−^ and reduce their recombination rates (Shaniba et al., 2020). Also, research has proved dopants can improve photocatalysis efficiency by reducing the requirement of absorbed energy, shifting absorbed light to visible wavelength, and enhancing photocatalysis activity (Ani et al., 2018; Yusuff et al., 2020). Some examples include nitrogen (Shaniba et al., 2020), pumice (Yusuff et al., 2020), Ag (Shahid et al., 2020), Ni (Serra, Zhang, et al., 2019), and Prussian blue (Rachna et al., 2020). Also, combining TiO_2_ or ZnO with other semiconductors to form heterojunction photocatalysts has been wildly studied, like black TiO_2_/g‐C_3_N_4_ (L. Shen et al., 2017) and ZnO/Bi_2_WO_6_ (Duan et al., 2020). Other metal compound photocatalyst materials also have been studied in the last several years, such as Bi_2_O_3_ (Zhang et al., 2020), MnO (Wang, Chen, et al., 2020), Ag_3_VO_4_ (Priyanka et al., 2020), and BiOBr (Jung et al., 2020).

In recent years, metal‐free carbon‐based materials have provoked much research interest. Some carbon‐based materials such as graphene, graphitic carbon nitride (g‐C_3_N_4_), and graphene oxide (GO) are nonmetal semiconductors and have been studied as cocatalysts due to their eco‐friendly composition, lightweight structure, high surface area, tunable band gap stability, and cost effectual process of preparation (Priyanka et al., 2020; Wang, Li, et al., 2020; Zheng et al., 2019). For instance, g‐C_3_N_4_ has 1.8–2.7 eV band gaps that allow the harvesting of visible light up to 460–698 nm (Zheng et al., 2016). Such metal‐free carbon‐based materials can combine to enhance photocatalytic ability, examples including g‐C_3_N_4_/GO aerogel (Tong et al., 2015) and g‐C_3_N_4_‐agar (Tan et al., 2019). The carbon‐based material also can combine with metallic compounds, such as sulfur‐doped graphene oxide (sGO)/Ag_3_VO_4_ (Priyanka et al., 2020) and Ag_3_PO_4_/polyaniline@g‐C_3_N_4_ (Balasubramanian et al., 2020). Some cutting‐edge carbon‐based materials also have been invested as photocatalysts, examples including carbon quantum dots (CQDs) (D. Choi et al., 2018; Rahbar et al., 2019), graphene quantum dots (GQDs) (Ge et al., 2016; Wei et al., 2016), and multiwalled carbon nanotubes (MWCNTs) (Cong et al., 2011; Yan et al., 2011). However, these materials only have been discovered in the last several decades, and the research on their green synthesis and mechanism is still at an early stage (Heng et al., 2021; Kaur et al., 2018)

There is less research on the homogenous catalyst, compared to heterogeneous catalysts. The homogenous catalyst usually combines light radiation with chemical oxidizing agents (Stan et al., 2012). PF is one of the most common homogenous catalysts, which usually consists of iron ion (Fe^2+/3+^) and hydrogen peroxide (H_2_O_2_) (Moncayo‐Lasso et al., 2009; Shwetharani & Balakrishna, 2014).

## METHODS OF SYNTHESIS

The synthesis of NP semiconductor materials is a major challenge in photocatalysis research. Synthesis methods have a significant impact on photocatalysts' morphology, structure, and performance (Sahu & Biswas, 2011; Sun et al., 2019; Taherinia et al., 2019).

### Metal‐associated visible light active photocatalysts synthesis

#### Sol‐gel method

The sol‐gel method has a large scope of application in the preparation of inorganic ceramic and glass materials (Czok & Golonka, 2016; Jones, 2013; Wang et al., 2014). Due to its wide range of advantages, including high chemical purity, good uniformity, and controllable morphology (Taherinia et al., 2019; Wetchakun et al., 2012), it has become a common method for photocatalysts synthesis. It is important to mention that a change in molar ratios may lead to different hydrolysis speeds and further different structures and properties (Lu et al., 2013; Wang et al., 2014; You et al., 2012).

**FIGURE 2:**
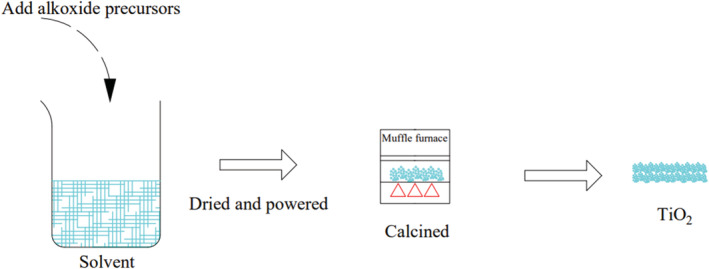
A typical sol‐gel method for TiO_2_

A typical example of the sol‐gel method is the synthesis of TiO_2_. In this method, TiO_2_ is synthesized by alkoxide precursors, like titanium butoxide (Ti(OBu)_4_) and titanium isopropoxide (TTIP). Initially, the sol‐gel method was a one‐step process (Long & Yang, 2002), and now, it usually contains two steps. First, dissolve the precursor with/without doping materials into a solvent, usually alcoholic (i.e., ethanol) or acid (i.e., HNO_3_). Due to hydrolysis, the sol is formed, and under polycondensation and action of density, the sol particles form a three‐dimensional network, resulting in the production of gel. Sonication could be applied in this step to enhance the reaction. Second, the gel is calcined in a muffle furnace at 400–700°C and, after this, it is dried and powdered (Kavil et al., 2020; Khataee et al., 2017; Taherinia et al., 2019; Villaluz et al., 2019). A schematic of this method is shown in Figure 2. Wang et al. (2014) have summarized the reaction mechanism as follows:

(13) $$\mathrm{Ti}\;{\left(\text{OR}\right)}_{n}+H_{2}O\to \mathrm{Ti}\left(\mathrm{OH}\right){\left(\mathrm{RO}\right)}_{n-1}+\mathrm{ROH}\;\left(\text{hydrolysis}\right)$$

(14) $$\mathrm{Ti}\left(\mathrm{OH}\right){\left(\text{OR}\right)}_{n-1}+H_{2}O\to \mathrm{Ti}{\left(\mathrm{OH}\right)}_{2}{\left(\text{OR}\right)}_{n-1}+\mathrm{ROH}\;\left(\text{hydrolysis}\right)$$

(15) $$–\mathrm{Ti}–\mathrm{OH}+\mathrm{HO}–\mathrm{Ti}–\to –\mathrm{Ti}–O–\mathrm{Ti}–+H_{2}O\;\left(\text{polycondensation}\right)$$

(16) $$–\mathrm{Ti}–\text{OR}+\mathrm{HO}–\mathrm{Ti}–\to –\mathrm{Ti}–O–\mathrm{Ti}+\mathrm{ROH}\;\left(\text{polycondensation}\right)$$

By adjusting the precursor and solvent, sol‐gel can also be used to synthesize other photocatalysts, examples including ZnO (Rafie et al., 2019), Bi_2_O_3_ (Zhang et al., 2020), Al_80_Ce_10_Zr_10_ (Perez‐Osorio et al., 2020), and Ag/g‐C_3_N_4_/V_2_O_5_ (El‐Sheshtawy et al., 2019).

#### Hydrothermal method

Hydrothermal technology is an important liquid‐phase preparation technology, which has extensive application in materials science, chemistry, physics, biology, metallurgy, and earth science (Yang & Park, 2019). It has been applied to produce nano TiO_2_ with high purity, crystal symmetry, metastable compounds with unique properties, and narrow particle size distributions (Byrappa & Adschiri, 2007) and is also preferred for ZnO synthesis (Danwittayakul et al., 2020). Concurrently, it has been widely used in photocatalyst synthesis, especially with carbon dopants (i.e., GO and activated carbon) (Shinde et al., 2018; Subramani et al., 2007; Yin et al., 2021; Zhang et al., 2017).

The hydrothermal method is a one‐step process. A typical example of its application is the synthesis of TiO_2_. By mixing a titanium precursor with water or NaOH/isopropanol/2‐propoanol(peptizer)–water solution, or alternatively TTIP with the acidic ethanol–water environment, with/without doping materials, add them into autoclaves for 16–72 h at 110–180°C. The precipitate is then calcined and dried to gain well‐defined TiO_2_ or composite TiO_2_ photocatalyst (X. Chen & Mao, 2007; Shinde et al., 2018)

The hydrothermal method can synthesize not only TiO_2_ and ZnO but also many other photocatalysts, including MoS_2_/reduced graphene oxide (rGO) (Gopalakrishnan et al., 2020), Mn_2_O_3_ (Zhao et al., 2017), ZnWO_4_ (Gong et al., 2020), FeCo_2_O_4_ (Yadav et al., 2019), and ZnS (Sabaghi et al., 2018).

#### Solvothermal method

The solvothermal method is very similar to the hydrothermal method, except the solvent used here is nonaqueous, with higher temperatures and pressures (X. Chen & Mao, 2007; Y. Wang et al., 2014). A typical procedure for using the solvothermal method to prepare ZnO is as follows. First, zinc acetate dehydrate (Zn(Ac)_2_·2H_2_O) or ZnCl_2_ is dissolved in organic solvent (i.e., ethanol and poly ethylene glycol), with/without doping materials. Then NaOH is added with ultrasonication mixing and transferred into an autoclave. After reaction and cooling, the ZnO is precipitated (Chouchene et al., 2016; Chu et al., 2014; Ruba et al., 2019). Apart from ZnO synthesis, the solvothermal method has a wide range of additional applications, including synthesizing TiO_2_ (Chavez et al., 2020), ZnFe_2_O_4_ (Ahmadpour et al., 2020), Ag_3_PO_4_/PANI@gC_3_N_4_ (Balasubramanian et al., 2020), MgFe_2_O_4_ (Y. Shen et al., 2013), and BiOI (Qiu et al., 2019).

#### Miscellaneous methods

The sol‐gel method, hydrothermal method, and solvothermal method are popular in the current study. Along with these commonly used synthesis methods, researchers have developed some additional methods and techniques.

Co‐precipitation can synthesize ZnO by adding solution (i.e., either adipic acid [C_6_H_10_O_4_]), ethylene glycol, or NH_4_OH into precursor aqueous solution (i.e., zinc acetate Zn(CH_3_COO)_2_·2H_2_O and Zn(NO_3_)_2_·6H_2_O), which create zinc adipate precipitation. After drying, zinc adipate is obtained by using both mediums and is decomposed at 450–500°C to produce ZnO (Thorat et al., 2012; Vlazan et al., 2015). Other applications include CuMgFe‐LDH (Zhang et al., 2020), magnetic graphene‐TiO_2_ (Chavez et al., 2020), and Bi_2_Ti_2_O_7_ (Y. F. Li, Zhong, et al., 2018).

The anodic oxidation method can be effectively employed to fabricate nanotubes and also has been developed for photocatalysts penetration (Nevarez‐Martinez et al., 2017; Tran et al., 2014). Examples include the V_2_O_5_‐TiO_2_ nanotube array (Nevarez‐Martinez et al., 2017) and MoS_2_/TiO_2_ nanotube arrays (Yan et al., 2020).

Solution combustion methods have also been developed by mixing precursors, fuel, and oxidizers under a high temperature to synthesize photocatalysts (Cubas et al., 2020; Meena et al., 2020). Some examples of materials developed include MFO/ZrO_2_ (Meena et al., 2020), Ag/CeO_2_ (Shang et al., 2020), and CuCrO_4_ (Cubas et al., 2020).

The template method entails the synthesis of monodisperse tubular or wire‐like nanostructures within the pores of a membrane or nanoporous solid (Wang et al., 2014). For example, Nozaki et al. (2018) used commercial silica microspheres as template material, using cerium(III) nitrate hexahydrate as a precursor to synthesize CeO_2_.

The flame aerosol method is a single‐step process and allows independent control of the material properties such as particle size, crystallinity, homogeneity, and degree of aggregation (Sahu & Biswas, 2011). In general, by burning precursors with fuel and oxygen, the photocatalyst will grow in the entrained air. Applications of this method include materials such as Cu‐TiO_2_ (Sahu & Biswas, 2011), C‐TiO_2_ (Lim et al., 2008), and Cr‐TiO_2_ (Inturi et al., 2014).

The reverse microemulsion method is used to prepare inorganic NPs (Zhou et al., 2011). There are two basic types of microemulsions: direct microemulsions (oil in liquid) and reverse microemulsions (liquid in oil) (Kar et al., 2018; Sautina et al., 2019). The continuous oil phase, surfactant, co‐surfactant, and precursor solution are prepared required for the reverse microemulsion system. After mixing, hydrothermal treatment, filtration, and drying, photocatalysts are prepared (Ge et al., 2010; Zhou et al., 2011). Some examples of materials developed include TiO_2_/sulfonated polyaniline (Zhou et al., 2011), Bi_2_WO_6_ (Ge et al., 2010), and CdS‐loaded K_4_Nb_6_O_17_ (Liang et al., 2013).

The sonochemical method has triggered much attention in advanced chemical synthesis as well as photocatalyst preparation. Ultrasonic generates alternating rarefaction and compression zones in this liquid, which leads to extremely high local energy densities (Wang et al., 2014). Briefly, precursors and capping agents reacted under ultrasonic and synthesized photocatalysts (Zinatloo‐Ajabshir et al., 2020). Examples of reactive materials developed through this technique include rGO‐V_2_O_5_ NPs (Mishra et al., 2020), silver tungstate (Zinatloo‐Ajabshir et al., 2020), and graphene oxide@ZnO (Muthukrishnaraj et al., 2020).

The chemical vapor deposition (CVD) method uses the chemical reaction of gaseous species to deposit thin solid films (Wang, Zhu, et al., 2020) and has been applied in photocatalyst film preparation. Examples include MoO_2_ film (Matamura et al., 2020), MoO_2_/MoS_2_/TiO_2_ sandwich heterostructure (Wang, Zhu, et al., 2020), and Fe_2_O_3_/2D graphene/Cu film (Polat, 2020).

The physical vapor deposition (PVD) method shares a similar application with the CVD method. It deposits films or forms coatings that are transported through a vacuum or low‐pressure gaseous/plasma environment (Wang et al., 2014), such as coating TiO_2_ (Shuang et al., 2016).

The electrodeposition method is a type of plating by depositing material on a substrate through electrochemical reduction (Hasan et al., 2019) and has been applied in photocatalyst preparation. Reactive materials developed using this method include Au/ZnO film (da Silva et al., 2013), TiO_2_ film (Hachisu et al., 2016), and TiO_2_ on a silicon wafer surface (Basheer et al., 2020).

Apart from these physical and chemical methods above, the biosynthesis process also has been developed. Ahmed et al. (2020) used A. Chinensis fruits extract with FeCl_2_·4H_2_O to prepare Fe NPs. Similarly, Ghazal et al. (2020) synthesize NiO NPs through the use of *Cydonia oblonga* extract.

### Carbon‐based visible light active photocatalysts synthesis

The calcination method has been used for graphitic carbon nitride (g‐C_3_N_4_) composite preparation. G‐C_3_N_4_ can be simply synthesized by directly calcining a precursor (i.e., melamine, urea, and thiourea) under an air atmosphere at around 550°C for several hours (L. Shen et al., 2017; Yu et al., 2016). Therefore, by calcining the mixture of melamine or urea and other materials, the g‐C_3_N_4_ composite can be synthesized (L. Shen et al., 2017; Yu et al., 2016; Zhao et al., 2018). Examples include black TiO_2_/g‐C_3_N_4_ (L. Shen et al., 2017), CeO_2_/g‐C_3_N_4_ (Yu et al., 2016), and CeVO_4_/g‐C_3_N_4_ (Ren et al., 2016).

Hummers's method (Hummers & Offeman, 1958) is commonly used in GO synthesis (Barakat et al., 2019; Mishra et al., 2020; Priyanka et al., 2020). It is an oxidation method treating graphite with essentially a water‐free mixture of concentrated sulfuric acid, sodium nitrate, and potassium permanganate to produce GO (Hummers & Offeman, 1958).

### Composite photocatalyst synthesis

Specific methods have been developed to synthesize composite photocatalyst. Many researchers do not synthesize common photocatalysts like TiO_2_ and ZnO in the laboratory but instead purchase them with specific specifications of surface area and particle size (Davididou et al., 2018; Jung et al., 2020; Sujatha et al., 2020; Vela et al., 2018c; Zuniga‐Benitez & Penuela, 2020). Methods introduced in previous sections may also apply to composite photocatalyst synthesis, but some unique methods are introduced below.

The wetness incipient impregnation method is used to synthesize composite photocatalysts by dissolving materials into the water followed by drying and calcining (Keshvadi et al., 2020; Yusuff et al., 2020). Examples include Ag‐CeO_2_ (Keshvadi et al., 2020), ZnO/pumice (Yusuff et al., 2020), and loading material on TiO_2_/ZeY (Roongraung et al., 2020).

The photodeposition method utilizes photocatalytic activity to prepare metal‐loaded photocatalysts. It uses photoexcited electrons to reduce metal cations to produce metal NPs on photocatalysts (Yamamoto et al., 2020). For example, Martins et al. (2019) mixed TiO_2_ (prepared by sol‐gel method) with Ag and Pd precursors in solvents, respectively, and under UV‐Vis light, Ag‐TiO_2_, and Pd‐TiO_2_ were synthesized. Other photocatalysts synthesized using a similar procedure include Fe_2_O_3_/TiO_2_ (Lee et al., 2017), Pt/TiO_2_ (Yamamoto et al., 2020), and TiO_2_‐Pd (Gmurek et al., 2019).

The chemical bath deposition (CBD) method is used for film or array photocatalyst preparation. Briefly, metal slides/flakes are placed in a chemical bath and photocatalytic deposition is encouraged forming a photocatalyst film or array. Specifically, the slides/flakes can be ordinary supporting inorganic material, like glass slides, or semiconductor material that involved photocatalysis, like TiO_2_ nanoflakes (Chai et al., 2014; Kite et al., 2020; Zhou et al., 2019). Examples include WO_3_/TiO_3_ array (Zhou et al., 2019), Zn_0.2_Cd_0.8_S films (Chai et al., 2014), ZnS thin films (Y. Chen et al., 2012), and FeSe thin films (Sohrabi & Ghobadi, 2019).

## WATER CONTAMINANT TREATMENT

### Textile and organic wastewater treatment

Textile wastewater often contains highly toxic and chemically stable organic compounds, including dyes, surfactants, oils, acid, alkali, solvents, and some metal salts, which are graded as one of the foremost pollutants in all industrial sectors (Ahmed et al., 2020; Kavil et al., 2020; Yusuff et al., 2020). Globally, it has been estimated that 280,000 tons of textile dyes alone are produced every year (Perez‐Osorio et al., 2020). Without appropriate treatment, before discharge, it has the potential to cause serious environmental contamination for receiving aquatic ecosystems.

#### TiO_2_ composites

TiO_2_ composites are a common form of photocatalyst, which have been widely investigated for textile and organic wastewater treatment. The commercial TiO_2_‐P25 already shows strong photocatalytic performance. Adamek et al. (2019) immobilized TiO_2_‐P25 on a glass fiber mat under sunlight and achieved decolorization of an anionic dye (Acid Orange 7; AO 7) within 20 min in comparison with commercial TiO_2_‐P25 alone.

The main purpose of modified TiO_2_ for visible light active photocatalysis is the enhancement of the visible light absorption capacity of the TiO_2_ composite. Lim et al. (2019) combined nanocubic‐like TiO_2_ with N‐doped graphene quantum dots (N‐GQDs) and proved N‐GQDs can provide light‐harvesting ability, especially in visible light and near‐infrared region, which completely removed BPA after 30 min. Similar results were also reported by Kavil et al. (2020) whereby a comparison of methylene blue (MB) removal was investigated from seawater against more conventional TiO_2_, C/TiO_2_, and Cu–C/TiO_2_ catalysts. The Cu–C/TiO_2_ had the best performance, which achieved maximum efficiency removing 100% MB in 45 min. Xu et al. (2020) prepared porous polymers/TiO_2_/Cu (PPTC) through a two‐step method and proved it performed better than TiO_2_ and TiO_2_/Cu. Feng et al. (2019) synthesized Z‐scheme Mn‐CdS/MoS_2_/TiO_2_ ternary photocatalyst, proving it had 3.16 times better performance than TiO_2_ alone when treating methyl orange (MO) and 9‐anthracenecarboxylic acids (9‐AC). It degraded *N,N*‐dimethylformamide (DMF), MO, MB, and phenol of 73.7%, 97.8%, 100%, and 98.6%, respectively, under 3 h of xenon light exposure. Gmurek et al. (2019) modified TiO_2_ catalysts with different metals (TiO_2_‐Pd, TiO_2_‐Au, TiO_2_‐Ag, and TiO_2_‐Pt) to treat five parabens (methylparaben [MP], ethylparaben [EP], propylparaben [PP], butylparaben [BuP], and benzylparaben [BeP]) and reported their performance under sunlight is better than under UVA. Among these TiO_2_ composites, TiO_2_‐Pd had the best performance, which totally degraded paraben in 120 min. Other composites' performance under sunlight and UV is shown in Table 3.

**TABLE 3:** TiO_2_ composites for textile and organic wastewater treatment

Photocatalyst	Synthesis method	Pollutants	Illumination	Result	Reference
TiO_2_‐P25 on the fiber mat	Dryer method	AO 7	Sunlight	Decolorization in 20 min	(Adamek et al., 2019)
N‐GQDs/TiO_2_	Hydrothermal and physical mixing methods	BPA	Sunlight	30 min, 100%	(Lim et al., 2019)
TiO_2_	Hydrolysis method	MB	Sunlight	60 min, 44%	(Kavil et al., 2020)
C/TiO_2_	Hydrolysis and sol‐gel methods			60 min, 100%	
Cu–C/TiO_2_				45 min, 100%	
PPTC	Hydrothermal and vacuum mixed methods	DMF, MO, MB, phenol	500 W Xe lamp	3 h, degradation rate: 73.7%, 97.8%, 100%, 98.6%	(Xu et al., 2020)
Z‐scheme Mn‐CdS/MoS_2_/TiO_2_ ternary	Hydrothermal method (Feng et al., 2017)	MO, 9‐AC	300 W xenon arc lamp	100 min, 98%; 35 min, 100%	(Feng et al., 2019)
TiO_2_‐Pd	Photodeposition method	Parabens	Sunlight	120 min, 100%	(Gmurek et al., 2019)
TiO_2_‐aluminum meshes	Sonochemical method (Barbosa et al., 2019; de Barros et al., 2014)	BR, TT	Sunlight	180 min, >99%	(Barbosa et al., 2020)
Fe/N/S‐TiO_2_	Sol‐gel method	4‐CP	Sunlight	180 min, 99.20%	(Villaluz et al., 2019)
SiO_2_/TiO_2_/calcined Liapor	Utility model (Jirkovsky et al., 2013)	4‐CP	Photoreactor with 10 fluorescent black lamps	360 min, 95%	(Bibova et al., 2019)
CNFT	Hydrothermal methods	MB	Sunlight	10 h, 100%	(Li, Hao, et al., 2018)
MgO@Ag_TiO_2_	Photodeposition method and ALD system (Zhao *et al*., 2017)	Phenol	150 W Oriel® Sol1A system	120 min, 95%	(Scott et al., 2019)
NFTS	Sol‐gel method	Basic red 29, Basic blue 41, Basic yellow 51	Sunlight with photoreactor	12 h, TOC removal rate: 58.5%	(Ghanbari et al., 2019)
WO_3_/TiO_2_ thin films	Spray pyrolysis and sonochemical method	BA	Sunlight	320 min, 66%	(Hunge, 2017)
TiO_2_/rGO/g‐C_3_N_4_ nanocomposite	Staudenmaier's (Moo et al., 2014) and calcination (Shen et al., 2017) methods	RTB	Sunlight	90 min, 60% decolorized, 51.43% degraded	(Das & Mahalingam, 2019)
CuCNBTNF	Photodeposition, thermal polymerization, and hydrogenating methods	RhB	150 W xenon lamp	5 h, 86.2%	(Choi et al., 2019)
NiS/RGO/TiO_2_	Hydrothermal method	TCP	Sunlight	6 h, 95%	(Alenizi et al., 2020)

The photocatalysts support material also has an impact on photocatalysis. Barbosa et al. (2020) supported TiO_2_ on aluminum mesh with H_2_O_2_ to degrade synthetic dyes Bordeaux Red (BR) and Tartrazine (TT) and achieve >99% degradation efficiency after 180 min. To remove 4‐chlorophenol (4‐CP), Bibova et al. (2019) coated SiO_2_/TiO_2_ composite on calcined Liapor, which showed calcined Liapor has the best removal rate among three lightweight substrates (natural cork, Liapor, and Sorbix), with the removal of around 95% in 360 min. Doped Fe/N/S on TiO_2_ (Fe/N/S‐TiO_2_) showed a better removal ability for 4‐CP, which achieved a 99.20% removal rate in 180 min (Villaluz et al., 2019). Similarly, S. Li, Hao, et al. (2018) employed a cellulose nanofiber/TiO_2_ aerogel (CNFT) to remove MB and achieve total degradation in 10 h.

To modify TiO_2_, many studies have applied metal materials. Scott et al. (2019) coated ultrathin MgO overlayer on Ag/TiO_2_ nanorods to treat phyenol and reported the thickness of MgO coating has a significant impact on photocatalysis. Under optimum conditions, the degradation efficiency is up to 95% in 120 min. Ghanbari et al. (2019) prepared a new N‐F‐codoped TiO_2_/SiO_2_ nanocomposite (NFTS) via the sol‐gel method to treat a mix of three azo dyes (Basic red 29, Basic blue 41, and Basic yellow 51). This achieved a 58.5% decrease in TOC under solar irradiation, in addition to reducing 100% of Cr(VI). Yuvaraj M. Hunge (2017) synthesized WO_3_/TiO_2_ thin films through spray pyrolysis and the sonochemical method, which degraded 66% benzoic acid (BA) after 320 min.

Carbon‐based material also has been applied for TiO_2_ photocatalysts. Das and Mahalingam (2019) immobilized TiO_2_, rGO, and g‐C_3_N_4_ in a polystyrene film under sunlight for the removal of Remazol Turquoise Blue (RTB), which achieved 60% decolorization and 51.43% degradation after 90 min. Also with g‐C_3_N_4_, J. U. Choi et al. (2019) prepared Cu‐loaded g‐C_3_N_4_/1D hydrogenated black TiO_2_ nanofiber (CuCNBTNF) for aqueous dye pollutant removal, which achieved 86.2% for Rhodamine B (RhB) in 5 h. Alenizi et al. (2020) synthesized nickel sulfide‐reduced graphene oxide‐titanium dioxide (NiS/RGO/TiO_2_) nanocomposite, which degraded 95% of trichlorophenol (TCP) in 6 h.

#### ZnO composites

ZnO composite photocatalysts are also applied for textile and organic wastewater treatment. The majority use metal materials to modify ZnO composites. For instance, Alseroury (2018) prepared ZnO/Mn_3_O_4_ to treat 4‐bromophenol (4‐BP) and 4‐CP, which achieved a >95% removal rate for both pollutants in 240 min. Similarly, Duan et al. (2020) used a two‐step hydrothermal method to synthesize ZnO/Bi_2_WO_6_ and proved that its photocurrent intensity is three times that of ZnO and Bi_2_WO_6_. Rafie et al. (2019) used Fe^3+^‐doped ZnO to treat Reactive Black 5 (RB5) bisazo dye, and the degradation rate was observed to be up to 98.32% within 3 h. Hu et al. (2019) doped Ce on ZnO rods via the one‐step pyrolysis method and found that 3% Ce exhibited the best RhB degradation rate, which degraded 97.66% within 2 h. Rachna et al. (2020) coupled Prussian blue (FeHCF) on ZnO through a solvothermal method to treat phenol, 3‐aminophenol (3‐AP), and 2,4‐dinitrophenol (2,4‐DNP) under sunlight and achieved the maximum 95%, 97%, and 93% degradation efficiency, respectively. Wei et al. (2019) prepared Fe_3_O_4_/ZnO/ZnS for MB removal, which removed 75.3% within 4 h. The Ag‐coated ZnO nanoflowers show a similar result with about 98.32% MB degradation efficiency (Shahid et al., 2020). Recently, Bora et al. (2022) synthesized Ag_2_CO_3_/ZnO via a simple precipitation route and reported it can achieve a totally TOC removal for MB wastewater in 30 min and 32% disinfection of *Escherichia coli* in an hour.

Along with the photocatalytic material, the structure of the photocatalytic material has a significant impact on photocatalysis performance. Serra, Zhang, et al. (2019) synthesized ZnO‐based biomimetic fern‐like microleaves and found ZnO@ZnS micro/nanoferns had the best photo‐remediation performance for persistent organic pollutants compared with ZnO, Ag‐ZnO, and Ni‐ZnO, which increased over sixfold for pollutant degradation rate capacity compared with pristine ZnO catalyst. The following research using the same bioinspired ZnO@ZnS photocatalyst achieved nearly 97% of MB after 60 min with mineralization of >98% of a mixture of MB, 4‐nitrophenol (4‐NP), and RhB after 210 min and the removal of nearly 65% of Cr(VI) after 180 min (Serra, Gomez, & Philippe, 2019). Recently, Bora et al. (2022) used a waste material‐ground granulated blast furnace slag (GGBFS) as a low‐cost geopolymer to hybridize with ZnO. They found that, due to the increased surface area, the discoloration efficiency of textile wastewater is twice better than normal ZnO (Table 4).

**TABLE 4:** ZnO composites for textile and organic wastewater treatment

Photocatalyst	Synthesis method	Pollutants	Illumination	Result	Reference
ZnO/Mn_3_O_4_	Chemical precipitation method	4‐BP, 4‐CP	Sunlight	240 min, >95%	(Alseroury, 2018)
ZnO/Bi_2_WO_6_	Hydrothermal method	Phenol, p‐chlorophenol, p‐nitrophenol	350 W xenon lamp	150 min, 99.3%, 100%, 100%	(Duan et al., 2020)
Fe^+3^‐doped ZnO	Microwave‐assisted sol‐gel method	RB5	72 W D65 lights	3 h, 98.32%	(Rafie et al., 2019)
Ce/ZnO	Pyrolysis method	RhB	Sunlight stimulator	2 h, 97.66%	(Hu et al., 2019)
ZnO@FeHCF	Solvothermal method	Phenol, 3‐AP, 2,4‐DNP	Sunlight	Degradation efficiency: 95%, 97%, 93%	(Rachna et al., 2020)
Fe_3_O_4_/ZnO/ZnS nanoparticles	Sol‐gel method	MO, MB	Sunlight	4 h, 79.5%, 75.3%	(Wei et al., 2019)
Ag‐coated ZnO nanoflowers	Template, hydrothermal, photoreduction methods	MB	Sunlight	3 h, removal rate: 98.32%	(Shahid et al., 2020)
ZnO/pumice	Incipient impregnation method	Effluent from Nike Art Gallery	Sunlight	45.04 min, degradation efficiency: 90.17%	(Yusuff et al., 2020)
Ag_2_CO_3_/ZnO	Precipitation	MB and *Escherichia coli*	Sunlight	0.5 h, 100% TOC removal 1 h, 32% disinfection	(Bora et al., 2022)
ZnO@ZnS core@shell	As‐electrodeposited method	MB, 4‐nitrophenol, RhB	UV‐filtered sunlight	120 min, ~99%	(Serra, Zhang, et al., 2019)
		MB; a mixture of MB, 4‐NP, and RhB; Cr (VI)	Sunlight	60 min, 97%; 210 min, >98%; 180 min, 65%	(Serra, Gomez, & Philippe, 2019)
GGBFS@ZnO	‐	Textile wastewater	Sunlight	Discoloration efficiency is twice better than normal ZnO	(Bora et al., 2022)

#### Other composites

Along with ZnO and TiO_2_ composites, other metallic oxides have been extensively investigated for their organic pollutant degradative capacity. For instance, to treat colored azo dye effluents, dos Santos et al. (2019) used Nb_2_O_5_ to treat MO, which achieved complete decolorization with H_2_O_2_ in 40 min. Kalin‐Fe_2_O_3_ had a similar performance, which removed 99.8% of RhB (Reddy et al., 2020). Abukhadra et al. (2018) synthesized bentonite/polyaniline composite (BE/PANI) as support for Ni_2_O_3_ (BE/PANI@Ni_2_O_3_), which reduced the band gap from 3.4 eV for pure Ni_2_O_3_ to only 1.61 eV, which totally removed safranin‐O dye after 90 min. Similarly, for ponceau SS dye, Jung et al. (2020) modified sodium vermiculite and achieved an 84.1% degradation rate in 360 min.

Besides metallic oxides, metallate photocatalysts also have been widely developed. Zinatloo‐Ajabshir et al. (2020) created nanostructured silver tungstate (Ag_2_WO_4_) by sonochemical pathway and reported that it can degrade 94.23% of Acid red 14 (AR14), 96.31% of eriochrome cyanine R (ECR), and 100% of RhB within 60 min. Similarly, an all‐day‐active photocatalyst was synthesized by employing Ag@AgI NPs decorated with Ag_3_PO_4_ cubes (C‐Ag_3_PO_4_@Ag@AgI) designed by Cai et al. (2019), which completely degraded RhB and removed 80% of BPA in 80 min under sunlight. They also reported that this photocatalyst can maintain photocatalytic activity even on a cloudy day. Bora and Mewada (2017) synthesized Ag_2_CO_3_/SiC through a simple precipitation route and found that SiC improves Ag_2_CO_3_ photoactivity by inducing a charge transfer between SiC and Ag_2_CO_3_ mimicking the Z‐scheme in photosynthesis, which removes 98% of MB in 4 h. X. Han et al. (2020) developed (Mg,Ni)(Fe,Al)_2_O_4_, which is a heterogeneous PF‐like catalyst that can degrade over 90.0% of common organic dyes within 180 min. Yadav et al. (2019) used nanoflower‐like FeCo_2_O_4_ to remove crystal violet (CV) and achieved a 94.19% removal rate in 160 min. Khan et al. (2019) electro‐deposited nickel nitrate powder [Ni(NO_3_)_2_·6H_2_O] inside the aluminum template to prepare one‐dimensional Ni nanorods to remove methyl red (MR) and MO. It achieved complete degradation in 40 and 60 min, respectively. For MB, Dong et al. (2019) used ZnSn(OH)_6_ nanocubes as the catalyst and, after 5 h of sunlight irradiation, achieved a 76.3% removal rate. Senthilnathan et al. (2019) deposited thin films of akaganeite (FeO(OH)) nanorices on muscovite mica surfaces (ANPM) and reported its MB degradation efficiencies under sunlight (89%) are better than under UV (87.5%). Better removal efficiency is achieved by MnFe_2_O_4_/ZrO_2_ nanocomposite, which removes 95% MB in 90 min (Meena et al., 2020). Ahmed et al. (2020) used nanosized iron (FeNPs) solely to remove lizarin yellow R (AYR) dye, and in 42 h, 93.7% of AYR was degraded. Y. M. Hunge et al. (2019) prepared Cu_2_ZnSnS_4_ (CZTS) for phthalic acid (PA) removal and achieved a 56% removal rate in 240 min. Mota et al. (2020) prepared Zn(II)‐porphyrin/poly(acrylic acid) (Zn(II)Pr@PAA) hybrid microparticles though and tested its performance by degrading MB, MO, and nitrobenzene (NB), which achieved above 96% chemical oxygen demand (COD) removal rate in 90 min. For azo dyes, Cubas et al. (2020) synthesized CuCr_2_O_4_ and removed 99.6% of it in 120 min.

Bismuth‐based compounds have been recognized as promising visible light‐responsive photocatalysts due to their unique layer structure and tremendous capacity for visible light absorption (Dutta et al., 2020; Q. Han, 2020; Z. J. Liu et al., 2019). Some investigations have been carried out recently to invest and enhance their performance. Z. J. Liu et al. (2019) constructed flower‐like BiOCl/BiOCOOH p‐n heterojunctions via an in situ anion‐exchange strategy and totally removed MO dyes in 50 min. Similarly, 3D flower‐like Ag/AgCl/BiOCOOH ternary heterojunction photocatalyst was used to remove RhB and CIP, which achieved 100% and 86.9% removal efficiency, respectively (S. J. Li, Xue, et al., 2019). They also prepared flower‐like Ag_2_CO_3_/BiOCOOH to remove RhB and MB, which achieved 100% removal efficiency in 30 min and 100% in 60 min (S. J. Li, Mo, et al., 2018). Jung et al. (2020) removed trichloroethylene (TCE) from the solution using BiOBr enhanced by sulfite addition and achieved dechlorination efficiency (R_dech_) by about 58%. Similarly, to treat perfluorooctanoic acid (PFOA), Sun et al. (2019) used a microwave solvothermal method to apply BiOCl with oxygen vacancies and its removal efficiency was 2.7 and 33.8 times higher than that of BiOCl fabricated by the conventional solvothermal and precipitation methods (Song et al., 2017). In addition, synthesis methods can also affect photocatalysts' performance. Y. F. Li, Zhong, et al. (2018) compared Bi_2_Ti_2_O_7_ photocatalytic ability prepared by co‐precipitation and solvothermal method and found that the Bi_2_Ti_2_O_7_ photocatalyst via co‐precipitation method had better performance (240 min, 92.8% removal). Bismuth‐based catalysts can also be modified with other compounds. T. Liu et al. (2014) compared a pure Bi_2_Mo_3_O_12_ sample with Bi_2_Mo_3_O_12_/MoO_3_, due to the excellent adsorption behavior of MoO_3_, Bi_2_Mo_3_O_12_/MoO_3_ showed an obviously enhanced photocatalytic activity, which removes 92% of MB.

**FIGURE 3:**
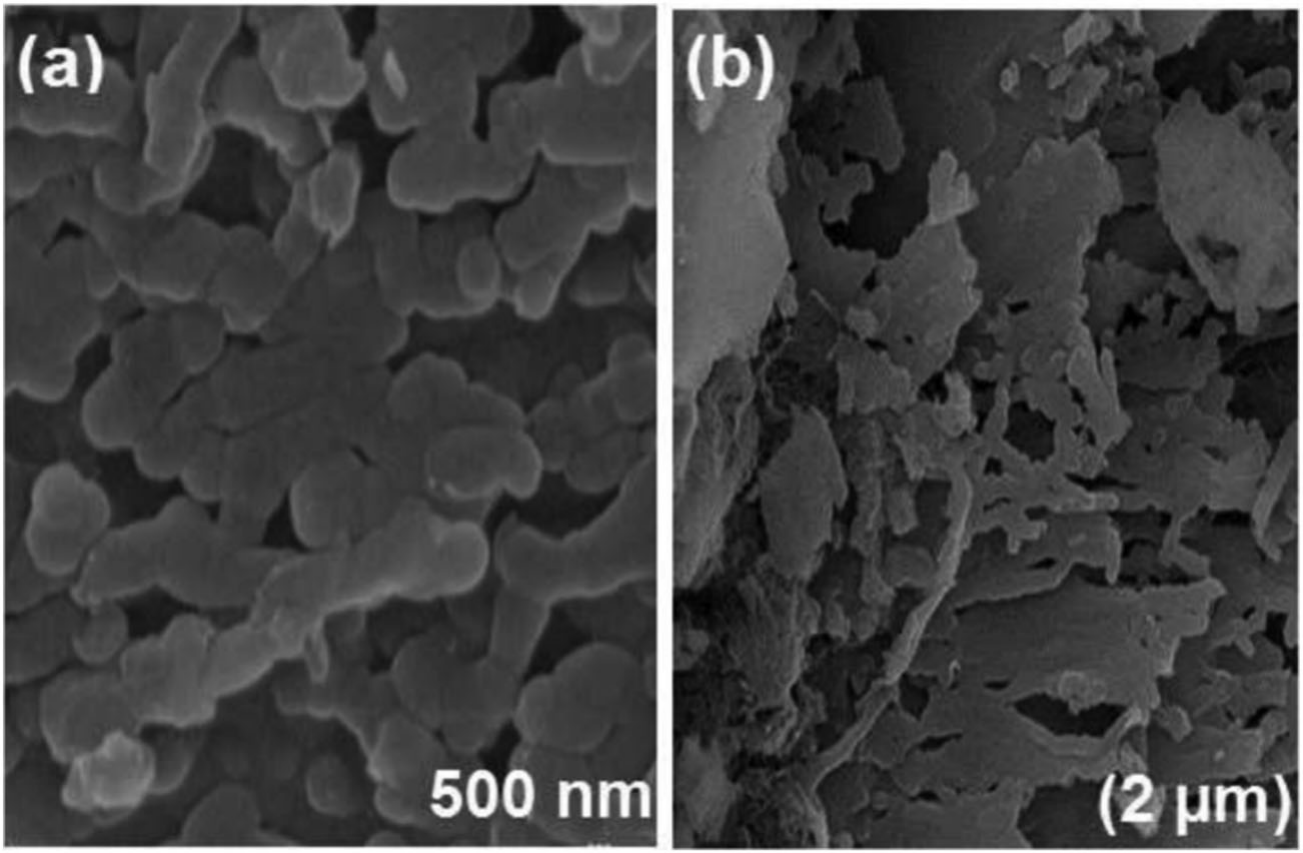
Schematic of the photocatalyst g‐C_3_N_4_ (Chegeni et al., 2019)

Carbon‐based materials also have a variety of applications in visible light active photocatalysis. Wang, Li, et al. (2020) synthesized sulfuric acid‐treated graphitic carbon nitride (SA‐g‐C_3_N_4_) by thermal polymerization and chemical exfoliation methods embedded within a porous cellulose network (CN/CA film) (T. Li et al., 2013; Xu et al., 2013), which removed ~99% RhB in 150 min and reduced 95% of Cr(VI) in 100 min. Mishra et al. (2020) used RGO‐V_2_O_5_ nanocomposite, which degraded 71% of MB in 20 min. A complete MB degradation was achieved by a hybrid of Zr‐based metal‐organic framework (UiO‐66) with graphitic carbon nitride (g‐C_3_N_4_) nanosheets (UiO‐66/g‐C_3_N_4_ sheets) photocatalysts within 240 min (Zhang et al., 2018). A similar result from V_2_O_5_/S‐g‐C_3_N_4_ was achieved by Chegeni et al. (2019) for MB and phenol treatment, which achieved a 99% and 89% removal rate within 60 min, respectively (Figure 3). Also, El‐Sheshtawy et al. (2019) immobilized Ag on g‐C_3_N_4_/V_2_O_5_ surface to enhance its photocatalytic activity, which can totally reduce p‐nitrophenol (NP) within 8 min under sunlight. They also reported that this photocatalyst only needs 60 min to totally reduce 4‐NP and 4‐AP in dark. Priyanka et al. (2020) synthesized sulfur‐doped graphene oxide (sGO/Ag_3_VO_4_/Ag) through Hummers's method (Hummers & Offeman, 1958) with a one‐pot method that can degrade above 99% cationic dyes, 75%–80% anionic dyes, and 90% organic carbon in 1 h under sunlight. Sharma et al. (2019) used activated carbon‐supported strontium/cerium bimetallic nanocomposite (Sr/Ce/AC BNC) to degrade RhB and reach 91% total degradation in 120 min. The wastewater mixture of MB and RhB was treated by MoS_2_/rGO/Cu_2_O grown on etched carbon paper, and 95% of MB and RhB are degraded in 45 min (Gopalakrishnan et al., 2020). The complete removal of MB was achieved by flexible graphene composites (FGCs) with Al_2_O_3_:Eu^3+^ and SrAl_2_O_4_:Bi^3+^ catalysts, respectively, after 180 and 270 min (Oliva et al., 2018).

Apart from the heterogeneous photocatalyst covered above, Sharmila et al. (2020) prepared mixed *Spirulina platensis* cultivated water (Spcw) as a homogeneous photocatalyst motivated by its benign chemical composition and eco‐friendly properties. This catalyst was shown to remove a mixture of 5 ppm of MB, 70 ppm of malachite green (MG), and 6 ppm of congo red (CR) within 3 h (Table 5).

**TABLE 5:** Other composites for textile and organic wastewater treatment

Photocatalyst	Synthesis method	Pollutants	Illumination	Result	Reference
Nb_2_O_5_	Calcined method	MO	Sunlight	100% decolorized	(dos Santos et al., 2019)
Kaolin‐Fe_2_O_3_	Sol‐gel method	RhB, CIP	Sunlight	60 min, RhB, 99.8%; COD, 88.3%	(Reddy et al., 2020)
BE/PANI@Ni_2_O_3_	In situ polymerization and oxidation (Dey et al., 2015) methods	Safranin‐O dye	Sunlight	90 min, 100%	(Abukhadra et al., 2018)
Modified sodium vermiculite	Cationic exchange with NaCl (Batista et al., 2019)	Ponceau SS dye	Sunlight	360 min, 84.1%	(Jung et al., 2020)
Ag_2_WO_4_	Sonochemical	RA14, ECR, RhB	125 W Osram lamp	60 min, removal rate: 94.23%, 96.31%, 100%	(Zinatloo‐Ajabshir et al., 2020)
C‐Ag_3_PO_4_@Ag@AgI	Ion‐exchange method	RhB, BPA	Sunlight	80 min, 100%; 80%	(Cai et al., 2019)
Ag_2_CO_3_/SiC	Precipitation	MB	Sunlight	4 h, 98%	(Bora & Mewada, 2017)
(Mg,Ni)(Fe,Al)_2_O_4_	Hydrochloric acid leaching (Yan et al., 2015) and co‐precipitation–calcination methods	Common organic dyes	Xenon lamp (with an AM 1.5 G filter)	180 min, 90.0%	(Han et al., 2020)
Nanoflower‐like FeCo_2_O_4_	Hydrothermal method (Yadav et al., 2018) with ultrasonic probe sonicator	CV	Sunlight	160 min, 94.19%	(Yadav et al., 2019)
Ni (NO_3_)_2_·6H_2_O	Electrodeposition method	MR, MO	Sunlight	40 min, 100%; 60 min, 100%	(Khan et al., 2019)
ZnSn (OH)_6_ nanocubes	Liquid precipitation and hydrothermal methods	MB	Sunlight	5 h, removal rate: 76.3%	(Dong et al., 2019)
ANPM	Hydrothermal method	MB	Sunlight	180 min, 89%	(Senthilnathan et al., 2019)
MnFe_2_O_4_/ZrO_2_ nanocomposite	Solution combustion method	MB, textile dye wastewater	Sunlight	90 min, degradation rate: 95%, 59%	(Meena et al., 2020)
FeNPs	Biosysthesis method	AYR	Sunlight	42 h, removal rate: 93.7%	(Ahmed et al., 2020)
CZTS	Sonochemical method (Gomes et al., 2017; Hunge, 2017)	PA	Sunlight	240 min, 56%	(Hunge et al., 2019)
Zn (II)Pr@PAA	Reverse emulsion method	MB, MO, NB	Sunlight	90 min, COD removal rate: 96%	(Mota et al., 2020)
CuCr_2_O_4_	Self‐combustion method	Azo dyes	125 W high‐pressure mercury lamp	120 min, 99.6%	(Cubas et al., 2020)
Flower‐like BiOCl/BiOCOOH p‐n heterojunctions	Solvothermal and in situ anion‐exchange methods	MO	300 W Xe lamp	50 min, 100%	(Liu et al., 2019)
3D flower‐like Ag/AgCl/BiOCOOH ternary heterojunction photocatalyst	Solvothermal, in situ precipitation methods, light reduction methods	RhB; CIP	300 W Xe lamp	30 min, 100%; 86.9%	(Li, Xue, et al., 2019)
Flower‐like Ag_2_CO_3_/BiOCOOH	Solvothermal method	RhB; MB	300 W xenon lamp	30 min, 100%; 60 min, 100%	(Li, Mo, et al., 2018)
BiOBr/sulfite	Hydrothermal method	TCE	100 W Xr arc lamp/reflector	120 min, R_dech_ 58%; 300 min, R_dech_ 57%	(Jung et al., 2020)
BiOCl	Microwave solvothermal method (Cui et al., 2018)	PFOA	300 W xenon lamp	8.5 h, 68.8%	(Sun et al., 2019)
Bi_2_Ti_2_O_7_	Co‐precipitation method	RhB	300 W xenon lamp	240 min, 92.8%	(Li et al., 2018)
	Solvothermal method			240 min, 74%	
Bi_2_Mo_3_O_12_/MoO_3_	Precursor suspension	MB	300 W Xe light with a 420 nm cutoff filter	90 min, 92%	[176]
SA‐g‐C_3_N_4_/CN/CA film	Thermal polymerization and chemical exfoliation methods	RhB; Cr (VI)	Sunlight	Removal rate: 150 min, 99%; 100 min, 95%	(Wang, Zhu, et al., 2020)
RGO‐V_2_O_5_	Hummer's and sol‐gel method	MB	40 W tube light	20 min, removal rate: 71%	(Mishra et al., 2020)
UiO‐66/g‐C_3_N_4_	Calcination and thermal method	MB	350 W xenon lamp	240 min, 100%	(Zhang et al., 2018)
V_2_O_5_/S‐g‐C_3_N_4_	Thermally method	MB; phenol	Sunlight	60 min, 99%; 89%	(Chegeni et al., 2019)
Ternary Ag/g‐C_3_N_4_/V_2_O_5_	Photodeposition method	4‐NP, 4‐AP	Sunlight	8 min, 100%	(El‐Sheshtawy et al., 2019)
SGO‐Ag_3_VO_4_/Ag	Hummer's method, the one‐pot method	Cationic dyes, anionic dyes, organic carbon	Sunlight	1 h, degradation rate: 99%, 75%–80%, 90%	(Priyanka et al., 2020)
Sr/Ce/AC BNC	Microwave reduction method	RhB	Sunlight	120 min, 91%	(Sharma et al., 2019)
MoS_2_/rGO/Cu_2_O	Chemical precipitation method	MB, RhB	Sunlight	45 min, 98%	(Gopalakrishnan et al., 2020)
FGCs/Al_2_O_3_:Eu^3+^	Casting method (Li et al., 2014)	MB	Sunlight	180 min, 100%	(Oliva et al., 2018)
FGCs/SrAl_2_O_4_:Bi^3+^				270 min, 100%	
Spcw	‐	MB, MG, CR	Sunlight	Removal rate: 100% in 2, 1, 3 h	(Sharmila et al., 2020)

### Pharmaceutical wastewater

Pharmaceutical wastewater typically contains persistent organic pollutants that contain a high concentration of organic matter, microbial toxicity, and high salt content, which is difficult to biodegrade, and municipal water resource recovery facility cannot treat pharmaceutical wastewater effectively (Ahmad et al., 2017; Guo et al., 2017; Keshvadi et al., 2020). Pharmaceuticals and their metabolites, even at low concentrations, have potentially fatal effects on natural ecosystems and human health (He et al., 2020; Keshvadi et al., 2020). For example, the occurrence of antibiotics in an aquatic environment can develop antibiotic resistance (Ben et al., 2019; Walsh, 2013; Wang, Chen, et al., 2020). In addition, it has been shown that 58%–68% of consumed common mild analgesic medicine acetaminophen (ACE) is released into the environment, which may transform to N‐acetyl‐p benzoquinone‐imine causing protein denaturation, lipid peroxidation, and DNA damage to organisms (Behravesh et al., 2020; Tobajas et al., 2017).

TiO_2_ composites are also common for pharmaceutical wastewater treatment. Shaniba et al. (2020) synthesized TiO_2_/nitrogen‐doped holey graphene (TiO_2_/NHG) nanocomposite via hydrothermal and calcination methods to treat antibiotic cefixime and achieve complete mineralization in 25 mg/L concentration within 90 min. Using similar catalysts involving nitrogen‐doped TiO_2_ (N‐TiO_2_), Keshvadi et al. (2020) treated carbamazepine (CBZ), diclofenac (DCF), and trimethoprim (TMP) under sunlight. The high removal efficiency of up to 100% was observed. Similarly, Martins et al. (2019) used Pd‐TiO_2_ and Ag‐TiO_2_ to treat the mixture of sulfamethoxazole (SMX), CBZ, and lorazepam (LRZ). Pd‐TiO_2_ showed the best performance with total compound degradation in 15 min, and both of them had better removal ability than pure TiO_2_. Sacco et al. (2019) prepared nitrogen‐doped TiO_2_ coupled with ZnS blue phosphors (NTZsP_PS) and removed >95% of the antibiotic ceftriaxone (CEF). Alternatively, Amini et al. (2019) used magnetic TiO_2_/SiO_2_/Fe_3_O_4_ nanocomposite doped with nitrogen (NTSF) and achieved a 96.32% degradation rate of naproxen (NPX). TiO_2_ also can combine with carbon‐based material to treat pharmaceutical wastewater. Cunha et al. (2019) synthesize TiO_2_/AC to treat benzodiazepine drugs via the impregnation method and reported that TiO_2_/AC10% (*w*/*w*) had the best removal efficiency of >97.5% within 60 min. Shinde et al. (2018) synthesized Pt‐rGO‐TiO_2_ to treat pharmaceutical pollutant β blocker Propranolol and showed a 20‐fold increase in removal (COD removal rate: 94%) under simulated solar light compared with TiO_2_ alone under UV exposure. Notably, the contaminant degradative performance of TiO_2_ composites may not always be better than a simple conventional TiO_2_ catalyst. Palma et al. (2020) treated antibiotic chloramphenicol (CAP) and paracetamol (N‐(4‐hydroxyphenyl)acetamide) by TiO_2_ and PbS/TiO_2_. The results showed that TiO_2_ alone can remove 5% more CAP in 240 min than the PbS/TiO_2_ composite and can completely remove paracetamol in 235 min whereas PbS/TiO_2_ only removes 93% in 240 min. Moreover, Behravesh et al. (2020) compared the removal ability between zeolite‐supported TiO_2_ (TiO_2_‐Z) and ZnO (ZnO‐Z) to degrade ACE and codeine and reported that ZnO‐Z has better removal efficiency.

Other metallate photocatalysts also have been invested to remove organic contaminants. To remove the antibiotic tetracycline hydrochloride (TC), Z. J. Liu et al. (2019) synthesized flower‐like BiOCl/BiOCOOH p‐n heterojunctions, which achieved 80.4% removal efficiency in 60 min. AgI/Bi_24_O_31_Cl_10_ shows a similar degradation efficiency for TC, which degrades 85.35% in 1 h. It also can reduce 78.26% of Cr(VI) within 1 h (B. S. Li, Lai, et al., 2019). A better removal ability was achieved by (Mg,Ni)(Fe,Al)_2_O_4_ heterogeneous PF‐like catalyst, which degraded 90% of TC within 180 min (X. Han et al., 2020), and S. J. Li, Mo, et al. (2018) used flower‐like Ag_2_CO_3_/BiOCOOH to remove TC, which achieved 93.4% degradation efficiency in 120 min. To degrade LOM (lomefloxacin), Zhang et al. (2020) fabricate Bi_2_O_3_/CuNiFe layered double hydroxide (LDH) composite photocatalyst through the co‐precipitation method, which removes 84.6% of LOM (10 mg/L) in 40 min. For antibiotic levofloxacin (LEV) treatment, Wang, Chen, et al. (2020) prepared MnO@MnOx microspheres via the solvothermal method, which achieves 98.1% degradation and 81.4% mineralization in 30 min. To remove antibiotic ofloxacin (OFX), Geng et al. (2019) prepared Co_3_(PO_4_)_2_/Ag_3_PO_4_ composites, which achieved 88.8% degradation efficiency in 5 min. Tie et al. (2019) employed N‐doped ZnS photocatalyst and achieved a 99% removal rate for metronidazole (MTR). To apply the principle of “waste may govern waste,” Domingues et al. (2020) used red mud considered waste from the metal production industry to treat the organic waste compounds of CBZ, LRZ, and SMX and achieved 58%, 62%, and 51% removal efficiency, respectively (Table 6).

**TABLE 6:** Photocatalysts for pharmaceutical wastewater

Photocatalyst	Synthesis method	Pollutants	Illumination	Result	Reference
TiO_2_/NHG	Hydrothermal and calcination	Cefixime	Sunlight	90 min, removal rate: 100%	(Shaniba et al., 2020)
N‐TiO_2_	Sol‐gel method (Sacco et al., 2012)	CBZ; DCF; TMP	Sunlight	247 min, 32%; 270 min, 100%; 300 min, 5%	(Keshvadi et al., 2020)
Pd‐TiO_2_	Sol‐gel and photodeposition (Gomes et al., 2017) methods	SMX, CBZ, LRZ	Sunlight	15 min, 100%	(Martins et al., 2019)
Ag‐TiO_2_				60 min, 100%	
NTZsP_PS	Sol‐gel method (Sacco et al., 2012)	CEF	Sunlight	150 min, >95%	(Sacco et al., 2019)
NTSF	Sol‐gel method	NPX	Sunlight	217.06 min, 96.32%	(Amini et al., 2019)
TiO_2_/AC	Impregnation method	Benzodiazepine drugs	OSRAM 300 W Ultra‐Vitaluz lamp	60 min, 97.5%	(Cunha et al., 2019)
Pt‐rGO‐TiO_2_	Hydrothermal method	β blocker Propranolol	1000 W xenon lamp	4 h, 94%	(Shinde et al., 2018)
TiO_2_	‐	CAP; paracetamol	Sunlight	240 min, 98%; 235 min, 100%	(Palma et al., 2020)
PbS/TiO_2_	Sulfide produced in the UAPB reactor			240 min, 93%; 93%	
TiO_2_‐Z	Solution mixing method (Hosseini et al., 2007)	ACE‐codeine	Sunlight	2 h, 39.2%	(Behravesh et al., 2020)
ZnO‐Z	Hydrothermal impregnation method (Byrappa et al., 2006)			2 h, 45.7%	
Flower‐like BiOCl/BiOCOOH p‐n heterojunctions	Solvothermal and in situ anion‐exchange methods	TC	300 W Xe lamp	60 min, 80.4%	(Liu et al., 2019)
AgI/Bi_24_O_31_Cl_10_	Solvothermal and in situ precipitation methods	TC; Cr (VI)	300 W xenon lamp	1 h, 85.34%; 78.26%	(Li, Lai, et al., 2019)
(CuC_10_H_26_N_6_)_3_(PW_12_O_40_)_2_/AgCl@Ag	In situ co‐precipitation method	TC; DNP	500 W Xe arc lamp	2 h, 85%; 65%	(Chen et al., 2019)
Flower‐like Ag_2_CO_3_/BiOCOOH	Solvothermal method	TC	300 W xenon lamp	120 min, 93.4%	(Li, Mo, et al., 2018)
Bi_2_O_3_/CuNiFe LDH	Co‐precipitation	LOM	35 W xenon lamp	40 min, 84.6% removal rate	(Zhang et al., 2020)
MnO@MnOx	Solvothermal	LEV	500 W xenon lamp	30 min, 98.1% degradation and 81.4% mineralization	(Wang, Chen, et al., 2020)
Co_3_(PO_4_)_2_/Ag_3_PO_4_	Hydrothermal method	OFX	300 W Xe lamp	5 min, 88.8%	(Geng et al., 2019)
N‐ZnS	Solvothermal method	MTR; CIP	Sunlight	150 min, 99%; 42%	(Tie et al., 2019)
G‐C_3_N_4_‐agar	Heating–cooling polymerization process	CIP; MB	500 W xenon lamp	180 min, 92%; >99%	(Tan et al., 2019)
Red mud	‐	CBZ; LRZ; SMX	Sunlight	1 h, 58%; 62%; 51%	(Domingues et al., 2020)

### Disinfection

Worldwide, it is estimated that 844 million people still do not have access to basic drinking water services, and 159 million people in rural areas use untreated drinking water, which potentially may expose them to health risks from contaminated water sources (World Health Organization & Unicef, 2017; Yan et al., 2020). For example, 4 billion cases of diarrhea each year are caused by inadequate hygiene and sanitation drinking water (Danwittayakul et al., 2020; World Health Organization & Unicef, 2015). Compared with traditional drinking water disinfection methods, like adsorption, coagulation, chemical, and physical disinfection, solar disinfection (SODIS) is much cheaper and easier to access in most areas, and it can be environmentally favorable to inactivate microorganisms in natural surface waters and drinking water (Garcia‐Gil et al., 2020; Malato et al., 2009; Rodriguez‐Chueca et al., 2019; Zeng et al., 2020). In recent years, photocatalytic disinfection has sparked much attention and established a trend to replace traditional solar disinfection (Djellabi et al., 2020; Vivar et al., 2020; Yan et al., 2020). Just like the mechanism of photocatalytic degradation of pollutants, photocatalysts can produce hydroxyl radical (·OH), h^+^, and superoxide radical (·O_2_^−^), which also can deactivate pathogens' destroying cell membrane structure to achieve disinfection (Ge et al., 2016; Zeng et al., 2020).

TiO_2_ composite catalysts are very common for water disinfection applications. Yan et al. (2020) used an anodic oxidation method to fabricate MoS_2_/TiO_2_ nanotube arrays and investigated its inactivation ability by treating *E. coli* ATCC 25,922 (*E. coli* 25922) and Methicillin‐resistant *Staphylococcus aureus* (MRSA). The result shows that the bacteria have been completely inactivated after 150 min with up to 98.5% disinfection efficiency. Fernández‐Ibáñez et al. (2015) developed TiO_2_‐rGO via a sonochemical method. Importantly, they proved that the concentration of TiO_2_‐rGO may not be proportional to disinfection efficiency. At 500 mg/L of TiO_2_, only 10 min of solar treatment can completely inactivate *E. coli*. On the contrary, the best *F. solani* inactivation efficiency was observed for 10 mg/L, requiring 30 min of treatment for complete inactivation. Waso et al. (2020) also used the same material by the same preparation method to treat rainwater and removed all *Klebsiella pneumoniae* (from 2.00 × 109 CFU/ml to below the detection limit [BDL]) with 120 min of natural sunlight exposure after pre‐treatment by *Bdellovibrio bacteriovorus*.

ZnO composites have also been extensively investigated. Danwittayakul et al. (2020) synthesized ZnO nanorods on cellulose and polyester substrates, which achieved nearly total disinfection within 15 min. A similar result was achieved by Yadav et al. (2019), where ZnO NPs were applied to enhance solar disinfection for fecal coliforms with compound parabolic concentrators. The result showed that there was complete inactivation within 15 min, which was a 50% improvement without using ZnO (Table 7).

**TABLE 7:** Photocatalysts for enhanced solar disinfection

Photocatalyst	Synthesis method	Pollutants	Illumination	Result	Reference
MoS_2_/TiO_2_	Anodic oxidation method	*Escherichia coli* 25922	300 W xenon lamp	150 min; 98.5%	(Yan et al., 2020)
TiO_2_/rGO	Sonochemical method	ATCC 23631, CECT 20232	Sunlight	Complete inactivation, 10 min, 30 min	(Fernández‐Ibáñez et al., 2015)
TiO_2_/rGO	Sonochemical method	*Klebsiella pneumoniae; Enterococcus faecium*	Sunlight	120 min, 100% removal rate; 240 min, 8.00 logs	(Waso et al., 2020)
ZnO	Hydrothermal method	*E. coli*	Sunlight	15 min, 97%–98%	(Danwittayakul et al., 2020)
ZnO	Sol‐gel method (Feng et al., 2011)	Fecal coliforms	Sunlight	15 min, 100%	(Yadav et al., 2019)

### Other application

Visible light active photocatalyst also applies in many other fields. In agriculture, to deal with the enormous use of pesticides, Balasubramanian et al. (2020) used Ag_3_PO_4_/polyaniline@g‐C_3_N_4_ to treat monocrotophos (MCP), a hazardous pesticide. By using the 150 W Xe arc lamp with a UV (λ > 400 nm) cutoff filter to simulate sunlight, it removed 99.6% MCP. To treat 2, 4‐dichlorophenoxy acetic acid (2, 4‐D), methyl chlorophenoxy propionic acid (MCPP), and 3, 6‐dichloro‐2‐methoxy benzoic acid (Dicamba), which are present in many of the shelf herbicide products, Heydari et al. (2019) used Buoyant titanium dioxide (TiO_2_)‐coated glass spheres and achieved 99.8%, 100%, and 99.4% removal rate, respectively. To treat chlorpyrifos (CP), thiamethoxam (TH), and tebuconazole (TEB), Rani and Shanker (2018) prepared metal hexacyanoferrate NPs (ZnHCF, CuHCF, NiHCF, and CoHCF), which all have direct low band gap, and the best degradation was achieved by ZnHCF (98% CP; 95% TH; and 91% TEB). Also, combining olive stone activated carbon (OSAC), ozone, and Solar‐Simulated Radiation (SSR) system has been reported can remove the pyridine‐based herbicides clopyralid completely (Rajah et al., 2019).

For urban wastewater treatment, Chavez et al. (2020) supported magnetite and TiO_2_ onto graphene combined with ozone and successfully treated the effluent of an urban wastewater treatment facility with 10 well‐known micropollutants. Similarly, Kaur et al. (2020) used rGO‐TiO_2_ to remove triclosan totally in actual urban wastewater. In marine conservation, Qiu et al. (2019) deposited the metal semiconductor (BiOI) on the expanded perlite (EP), which is able to degrade 86% of diesel‐contaminated seawater within 2 h.

To reduce Cr(VI), Zhang et al. (2019) synthesized Iron(III)‐alginate (Fe‐SA) hydrogel beads, which reduced Cr(VI) up to 100% within 150 min. Nitrogen–phosphorus‐doped fluorescent carbon dots (NP‐CD) show high efficiency for Cr(VI) in a linear range from 10 ppm (in approximately 10 min) to 2000 ppm (in approximately 320 min) (Bhati et al., 2019). To treat nitrate (NO_3_^−^‐N) and ammonia (NH_4_^+^‐N), Zhao et al. (2018) synthesized 3D/2D Mn_2_O_3_/g‐C_3_N_4_ and achieved high removal efficiency of 94.5% and 97.4% for NO_3_^−^‐N and NH_4_^+^‐N (Table 8).

**TABLE 8:** Photocatalysts for other applications

Photocatalyst	Synthesis method	Pollutants	Illumination	Result	Reference
Ag_3_PO_4_/polyaniline@g‐C_3_N_4_	Calcination method (Zhang et al., 2018)	MCP	150 W Xe arc lamp with an ultraviolet (λ > 400 nm) cutoff filter	50 min, 99.6%	(Balasubramanian et al., 2020)
TiO_2_	‐	2, 4‐D, MCPP, Dicamba	Sunlight	99.8%, 100%, 99.4%	(Heydari et al., 2019)
ZnHCF	*Sapindus mukorossi* (Jassal et al., 2015)	CP; TH; TEB	Sunlight	12 h, 98%; 95%; 91%	(Rani & Shanker, 2018)
CuHCF				12 h, 91%; 89%; 85%	
NiHCF				12 h, 85%; 78%; 73%	
CoHCF				12 h, 83%; 76%; 70%	
O_3_/OSAC/SSR	Najar's method (Ouederni et al., 2006)	Clopyralid	500 W Xe lamp	30 min, removal rate: 100%	(Rajah et al., 2019)
S‐rGO/ZnS	Hummer's and hydrothermal method	‐	Sunlight	Enhanced the solubilized chemical oxygen demand by 113% after 6 h	(Barakat et al., 2019)
10‐MG1‐Ti	Solvothermal method (Cao et al., 2015)	Cotinine, caffeine, ciprofloxacin, metroprolol, sulfamethoxazole, *N*,*N*‐diethyl‐*m*‐toluamide, clofibric acid, bezafibrate, tritosulfuron, ibuprofen	Xe arc lamp	2 h, 70% TOC removal efficiency	(Chavez et al., 2020)
rGO–TiO_2_	Hydrothermal method (Marcano et al., 2010)	Triclosan	Sunlight	24 h, removal rate: 100%	(Kaur et al., 2020)
BiOI/EP	Solvothermal method	Diesel	300 W Xe lamp	2 h, 86%	(Qiu et al., 2019)
Fe‐SA	Sonochemical method (Titouhi & Belgaied, 2016)	Cr (VI) and As (III)	300 W xenon lamp	150 min, 100%	(Zhang et al., 2019)
NP‐CD	Microwave‐assisted method	Cr (VI)	Sunlight	A linear range	(Bhati et al., 2019)
Mn_2_O_3_/g‐C_3_N_4_	Calcination method	NO_3_^−^‐N; NH_4_^+^‐N	300 W xenon lamp	120 min, 94.5%; 97.4%	(Zhao et al., 2018)

## PILOT‐SCALE TESTS

### Current research

**FIGURE 4:**
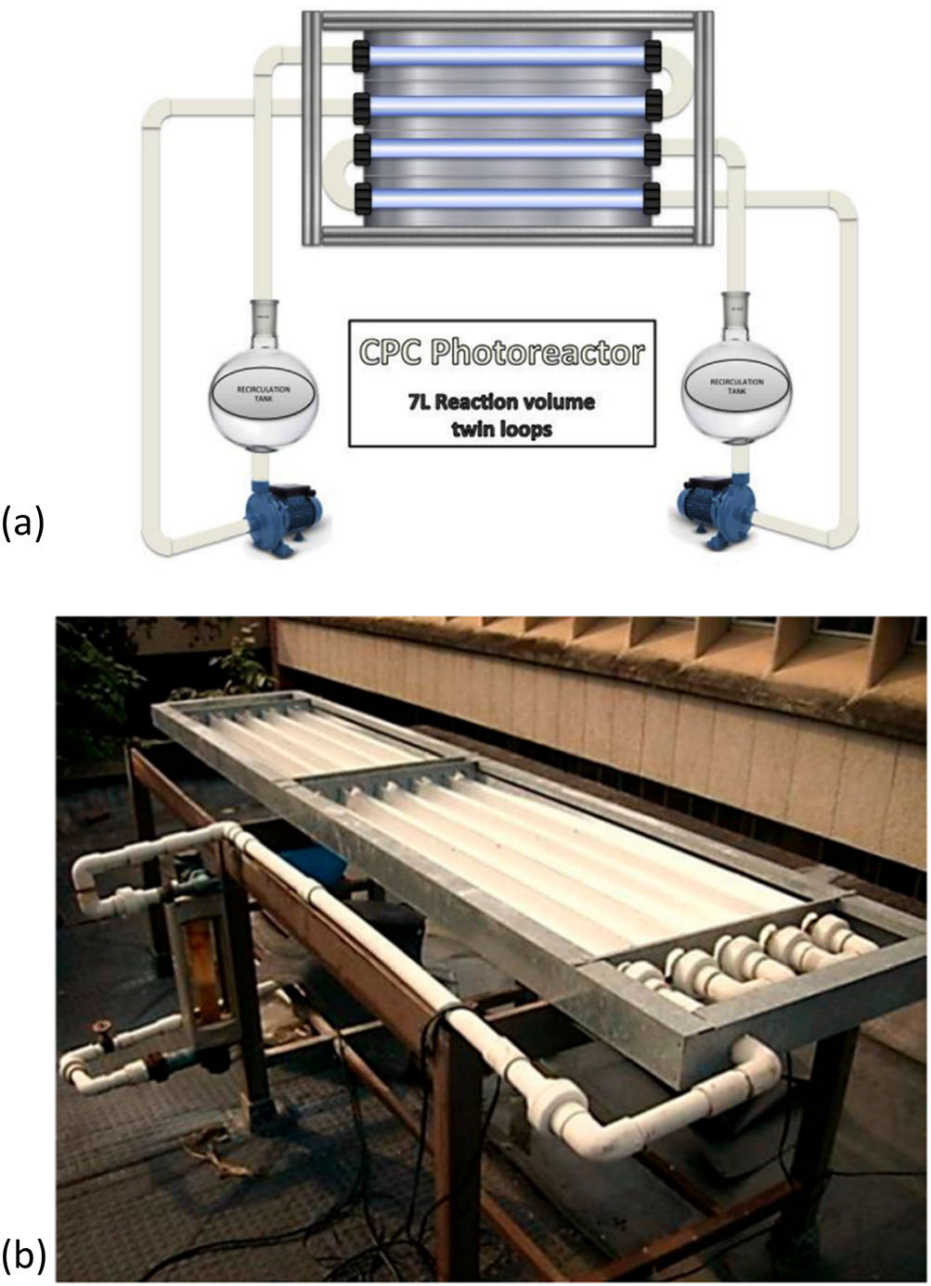
(a) Schematic of Compound Parabolic Collector (CPC) (Cabrera‐Reina et al., 2019); (b) photograph of CPC ready for application (Arce‐Sarria et al., 2018)

**FIGURE 5:**
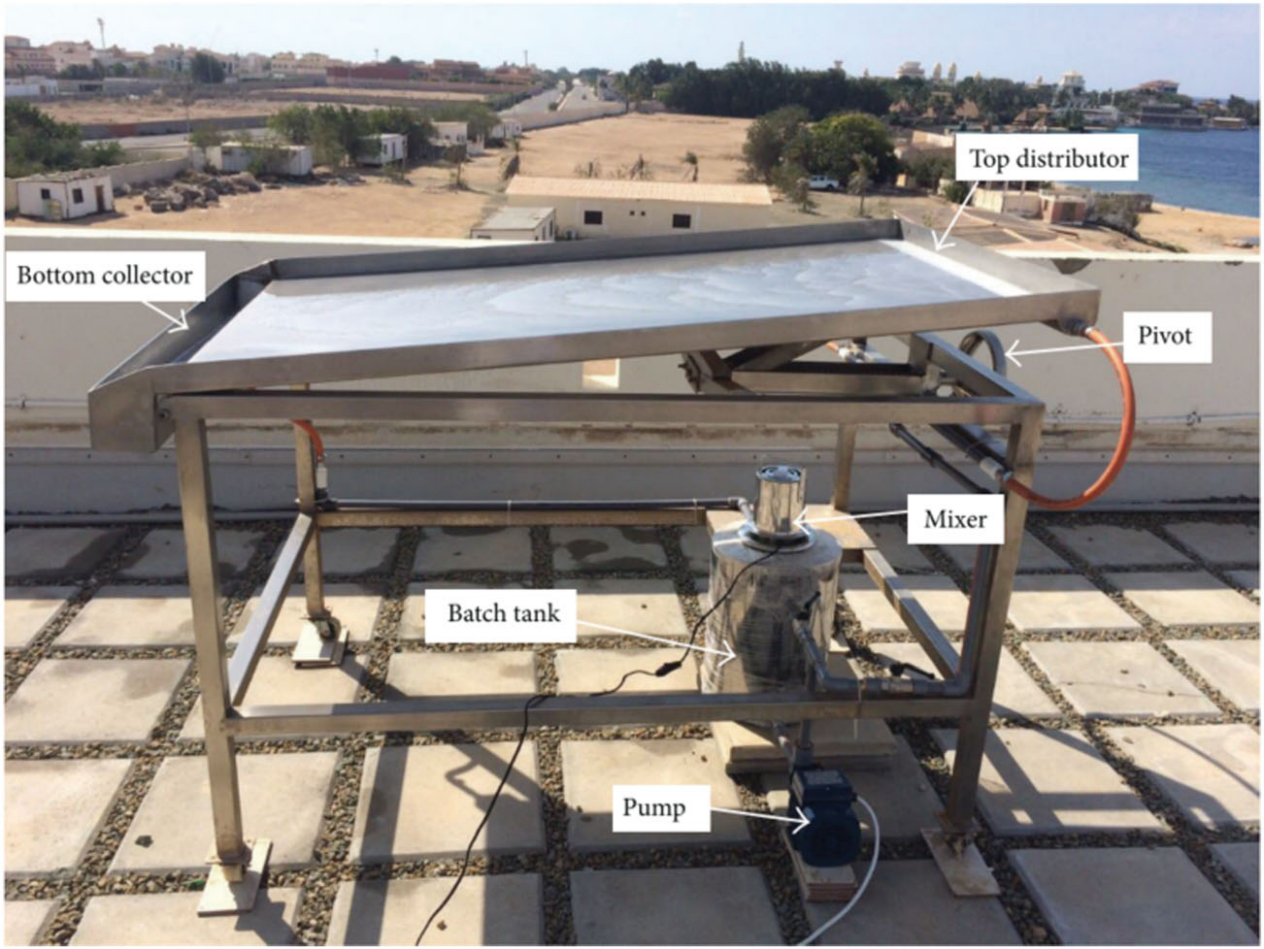
Picture of a solar falling film reactor (SFFR) (Shaban et al., 2016)

Over the past number of decades, many studies have invested in visible light active photocatalyst for water treatment. However, only a few of them have entered the pilot‐scale test. Based on the Scopus database, only approximately 23 papers have been published about pilot‐scale tests in water treatment using visible light/solar active photocatalysts, among them only 14 papers in the past 5 years (2018–2022). In this section, this research that has been successfully tested on a pilot scale will be introduced. Compound Parabolic Collector (CPC) (Figure 4) is widely applied in many photocatalyst pilot‐scale tests. Other equipment like falling film photoreactor (FFR) (Figure 5), tubular reactor, and self‐made reactor are also applied.

Majority of pilot tests carried out in the last 5 years used TiO_2_ with its composites as the photocatalyst. Just using the commercial TiO_2_ with CPC, Zuniga‐Benitez and Penuela (2020) tested their photodegradation ability for Benzophenone‐1 and Benzophenone‐2 (BP1 and BP2). The pilot scale was tested in the city of Medellín, Colombia, using a solar compound cylinder‐parabolic collector; greater than 90% of BP have been removed after 6 h of reaction (between 10:00 and 16:00 h). Using TiO_2_ with CPC for carbapenem antibiotics imipenem and meropenem degradation, they can remove 75% of imipenem after 60 min and 75% of meropenem after 45 min (Cabrera‐Reina et al., 2019). For degradation of diphenhydramine hydrochloride (DPH), Lopez et al. (2018) achieved 14,889 mg TOC/kWh demineralization efficiency but under black blue lamps has better efficiency (21,141 mg TOC/kWh). On the contrary, also using TiO_2_‐P25, Haranaka‐Funai et al. (2017) reported that the CPC has the best removal performance for valproic acid sodium salt (VA) treatment (91% VAC removal efficiency and 50% TOC degradation rate) compared with three artificial irradiation (black light blue lamps, UVC, and Xe lamp). Also applied CPC and TiO_2_‐P25, Grilla et al. (2019) found that the TMP degradation is proportional to SPS concentration and inversely proportional to water matrix complexity. Diaz‐Angulo et al. (2020) also used them to remove DCF; by applying MR as the photo‐sensitizer, the mineralization rate can be increased up to 65%, which removes 99% of DCF and 99% of MB.

Also applied CPC, to treat pesticides in wastewater, Vela et al. (2018b) used two commercial TiO_2_ nanopowders (Degussa P25 and Kronos vlp 7000) for degradation of a mixture of six pesticides (malathion, fenotrothion, quinalphos, vinclozoline, dimethoate, and fenarimol). After optimized operational conditions under laboratory conditions (Vela et al., 2015), they set up a polite scale test at Murcia, SE Spain (3000 h sun per year). The result showed that the use of TiO_2_ alongside an electron acceptor like Na_2_S_2_O_8_ can strongly enhance the degradation rate and the best result was achieved by TiO_2_ Kronos vlp 7000 with Na_2_S_2_O_8_, which achieved 90% degradation (DT_90_) in 32 min. These photocatalysts also have been tested to treat endocrine disruptors (EDs) at a pilot plant scale. Different from pesticides test results, TiO_2_‐P25/Na_2_S_2_O_8_ has the best performance, which achieved a 3 J/cm^2^ half‐fluence (H_50_). They also found that TiO_2_ (mainly P25) in tandem with Na_2_S_2_O_8_ can avoid recombination of e^−^/h^+^ pairs (Vela et al., 2018c). Except for TiO_2_, they also used ZnO/Na_2_S_2_O_8_ at the pilot plant scale. It can achieve an 83% removal rate for dissolved organic carbon (DOC) for EDs removal (Vela et al., 2018a) and min DT_90_ for several fungicides and insecticides removal (Vela et al., 2019). Some similar pilot‐scale tests using TiO_2_/ZnO with Na_2_S_2_O_8_ also have been completed (Fenoll et al., 2019; Kushniarou et al., 2019).

TiO_2_ composites have also been invested under pilot tests. For carbon‐based TiO_2_ composites, Luna‐Sanguino et al. (2020) synthesized two TiO_2_‐rGO from commercial TiO_2_ (P25 and Hombikat UV100, HBK) for several pesticides' treatments (methomyl, pyrimethanil, isoproturon, and alachlor) and run a pilot‐scale test in a 3.2 m^2^ CPC located in the Plataforma Solar de Almería (PSA‐CIEMAT). The TiO_2_‐rGO (P25) has a better performance, which removes all pesticides in 210 min. They also reported that the use of H_2_O_2_ can speed up the removal. Also, TiO_2_/H_2_O_2_ has been proved efficient for *Curvularia* sp. deactivation by using CPC, which achieved completed disinfection in 300 min (Aguas et al., 2017). Shaban et al. (2016) used carbon‐modified titanium oxide (CM‐n‐TiO_2_) to treat polychlorinated biphenyls (PCBs) in seawater on a pilot‐plant scale by a solar falling film reactor (SFFR), which reached completed degradation after 75 min. Vatanpour et al. (2019) modified TiO_2_ by urea for Reactive Orange 29 (RO29) removal and tested it in a continuous pilot‐scale submerged photocatalytic membrane reactor (SPMR). They found that modified TiO_2_ using urea with a 1:6 ratio (TiO_2_:urea) under 450°C can achieve 84.2% decolorization efficiency. Recently, Mesa et al. (2021) compared commercial TiO_2_ with UV/H_2_O_2_ in a 120 L CPC for effluents from handicraft factories. Their result showed that UV/H_2_O_2_ is the best treatment for dye and TiO_2_ achieved a better elimination of coliform bacteria. It is important to mention that TiO_2_ showed a detrimental effect on the overall elimination of dyes.

Ahmadpour et al. (2020) compared ZnFe_2_O_4_@TiO_2_/Cu nanocomposites removal ability for recalcitrant drug NPX in batch and large‐scale systems. In a large‐scale experiment, 1000 ml of NPX pollutant solution was treated and reported a 63.14% degradation rate in 120 min, compared with 80.73% for the batch experiment. After optimizing the photocatalyst design in a laboratory‐scale experiment, Bibova et al. (2019) designed two pilot‐scale tests for SiO_2_/TiO_2_/calcined Liapor. One is in Czech Republic to simulate the remediation of contaminated water in rural areas, and another is in Vietnam for a comparative solar experiment. In the Czech Republic, oxalic acid (OA; 3.3 × 10^−3^ mol/dm^3^) is chosen as a model compound and achieved an 82.1% TOC removal rate in 3 days. In Vietnam, MB as a model compound is used and achieved around 58% degradation in 6 days. Graywater is a highly reclaimable water source. Saran et al. (2019) prepared TiO_2_‐Ag for a pilot‐scale slurry‐type tubular photocatalytic reactor to treat actual graywater, with the addition of H_2_O_2_ a 99% COD abatement was achieved within 2 h. Tsoumachidou et al. (2017) used commercial TiO_2_‐P25 with H_2_O_2_ and Fe^3+^ to treat graywater, which achieved an almost 64% DOC removal rate in a pilot‐scale slurry fountain photoreactor. Olga Sacco et al. (2018) set a pilot‐scale test for real wastewater disinfection and MB removal using nitrogen‐doped TiO_2_ particles (N‐TiO_2_/PS) immobilized on polystyrene spheres. MB removal pilot‐scale test showed a similar result to the laboratory‐scale test, which achieve a 90% removal rate in 180 min. However, the disinfection pilot‐scale test showed a much worse result, which only inactivates 25% of *E. coli* in 120 min. Mohsenzadeh et al. (2019) used the composition of polyaniline (PAni) and TiO_2_ on glass beads to remove 1,2‐dichloroethane (1,2‐DCE) in a pilot‐scale packed bed recirculating photocatalytic reactor, which achieves complete degradation after 360 min. Saran et al. (2018) tested TiO_2_‐Ag for rainwater disinfection using a pilot‐scale solar photocatalytic fixed bed tubular reactor and achieved complete disinfection in addition to COD removal within 120 min. Y. Li et al. (2016) fabricated foam concrete incorporated with nitrogen‐doped TiO_2_ (N‐TiO_2_/FC) and tested its removal performance for trichlorfon in a pilot‐scale experiment, which reported that 6% N‐TiO_2_/FC can remove almost 100% of trichlorfon in 2 weeks. However, the TiO_2_ composite is not always better than commercial TiO_2_. Arce‐Sarria et al. (2018) modified TiO_3_ by WO_3_ and tested its photocatalytic performance for antibiotic amoxicillin by CPC, which achieved maximum 64.4% removal rate but still underperformed compared with the commercial TiO_2_‐P25.

Except for the TiO_2_ composite, some other composites also have been invested under pilot‐scale tests. For example, Dhatshanamurthi et al. (2017) coated ZnO on fevicol and found that it is more efficient than the catalysts made by commercial ZnO and TiO_2_‐P25 catalysts for really dye industry effluent treatment in a pilot‐scale FFR, which achieved around 95% COD removal rate in 4 h. Booshehri et al. (2017) synthesized Ag‐modified BiVO_4_ for *E. coli*, *E. faecalis*, and spores of *F. solani* inactivation and tested its photocatalytic disinfection by CPC, which achieved almost total degradation for *E. coli* after around 160 min, although its performance is still worse than TiO_2_‐P25 (Table 9).

**TABLE 9:** List of some recent pilot‐scale visible light active photocatalysts systems

Photocatalyst	Synthesis method	Pollutants	Illumination	Location	Result	Reference
TiO_2_‐P25	‐	BP	CPC	Medellín (latitude 6°13′51″, longitude 75°35′26″)	6 h; 90% removal rate	(Zuniga‐Benitez & Penuela, 2020)
TiO_2_‐P25	‐	Imipenem; meropenem	CPC	Solar Energy Research Center, Almería, Spain	60 min, 75%	(Cabrera‐Reina et al., 2019)
TiO_2_‐P25	‐	DPH	CPC	University of Barcelona (41.4°N, 2.1°W)	14,889 mg TOC/kWh	(Lopez et al., 2018)
TiO_2_‐P25	‐	VA	CPC	University of Barcelona (41.4°N, 2.1°W)	5 h, 91%	(Haranaka‐Funai et al., 2017)
TiO_2_‐P25 SPS	‐	TMP	CPC	Plataforma Solar de Almeria	Complete degradation	(Grilla et al., 2019)
TiO_2_/MB	‐	DCF	CPC	Iwaki centrifugal pumps	2 h, 99%	(Diaz‐Angulo et al., 2020)
TiO_2‐_P25/Na_2_S_2_O_8_	‐	A mixture of six pesticides	Sunlight	Murcia, SE Spain (37°59′N, 1°08′W)	DT_90_ varied from 79 to 1270 min	(Vela et al., 2018b)
TiO_2_ vlp 7000/Na_2_S_2_O_8_	‐	EDs	Sunlight	Murcia, SE Spain	DT_90_ varied from 32 to 817 min	(Vela et al., 2018c)
TiO_2_ P25/Na_2_S_2_O_8_	‐				H_50_: 3–58 J/cm^2^	
TiO_2_ vlp 7000/Na_2_S_2_O_8_	‐		Sunlight		H_50_: 10–117 J/cm^2^	
Commercial TiO_2_	‐	Effluents from handicraft factories	Sunlight	Nobsa, Boyacá, Colombia, coordinates 5°46′11″N, 72°56′24″O	7 h, 70.80% of the discoloration	(Mesa et al., 2021)
ZnO/Na_2_S_2_O_8_	‐	EDs	Sunlight	Murcia, SE Spain	240 min, DOC: 83%	(Vela et al., 2018a)
ZnO/Na_2_S_2_O_8_	‐	Vinclozoline; fenarimol; quinalphos; malathion; fenitrothion; dimethoate	Sunlight	Murcia, SE Spain	DT_90_: 237 min; 1001 min; 250 min; 26 min; 28 min; 72 min	(Vela et al., 2019)
TiO_2_‐rGO (P25)	Hydrothermal method	Methomyl, pyrimethanil, isoproturon, and alachlor	CPC	PSA‐CIEMAT	210 min, 100%	(Luna‐Sanguino et al., 2020)
TiO_2_‐rGO (Hombikat UV100)					240 min, 100%	
TiO_2_/H_2_O_2_	‐	*Curvularia* sp.	CPC	Spain	30 min, 100%	(Aguas et al., 2017)
CM‐n‐TiO_2_		PCBs	FFR	Jeddah	75 min, 100%	(Shaban et al., 2016)
TiO_2_/urea	Taguchi methods	RO29	SPMR with five 36 W visible lamps	‐	5 min, 90.2%	(Vatanpour et al., 2019)
ZnFe_2_O_4_@TiO_2_/Cu	Solvothermal (Gu et al., 2016), sonochemical, and Chi's (Chi et al., 2013) methods	NPX	Sunlight	‐	120 min, 63.14%	(Ahmadpour et al., 2020)
SiO_2_/TiO_2_/calcined Liapor	Utility model (Jirkovsky et al., 2013)	OA	Sunlight	Czech Republic	3 days, TOC removal rate: 82.1%	(Bibova et al., 2019)
		MB		Vietnam	6 days, 58%	
TiO_2_‐Ag	Photoreduction methods (Ubonchonlakate et al., 2012)	Graywater	Slurry‐type tubular photocatalytic reactor	Pondicherry University (12.01°N, 79.85°E)	2 h, 99% COD	(Saran et al., 2019)
TiO_2_/H_2_O_2_/Fe^3+^	‐		Pilot‐scale slurry fountain photoreactor	‐	210 min, 64% DOC	(Tsoumachidou et al., 2017)
N‐TiO_2_/PS	Solvent cast method	MB	Sunlight	Outside the Laboratory of Sanitary and Environmental Engineering, University of Salerno (40°N, 14°E)	120 min, 91% discoloration, 180 min, 90% TOC removal rate	(Sacco et al., 2018)
		*Escherichia coli*			120 min, 25%	
PAni‐TiO_2_	In situ deposition oxidative polymerization method	1,2‐DCE	Pilot‐scale packed bed recirculating photocatalytic reactor with 150 W xenon lamp	‐	360 min, 100%	(Mohsenzadeh et al., 2019)
TiO_2_‐Ag	Sol‐gel method	Microorganisms	Fixed bed tubular reactor under sunlight	Pondicherry University (12.01°N and 79.85°E)	120 min, 100%	(Saran et al., 2018)
N‐TiO_2_/FC	Aforementioned mixing procedure	Trichlorfon	Sunlight	‐	2 weeks, 100%	(Li et al., 2016)
TiO_3_‐WO_3_	Sol‐gel method (Ramos‐Delgado et al., 2013)	Amoxicillin	CPC	Cali, Colombia	Maximum 64.4%	(Arce‐Sarria et al., 2018)
Nano ZnO	Precipitation–thermal decomposition method (Velmurugan & Swaminathan, 2011)	Dye industry effluent	FFR	Annamalai University	4 h, 95%	(Dhatshanamurthi et al., 2017)
Ag/BiVO_4_	Homogeneous precipitation and photodeposition methods (Booshehri et al., 2014)	*E. coli*; *E. faecalis*; *F. solani*	CPC	Plataforma Solar de Almeria (Almería, Spain) (37°84′N, 2°34′W	160 min, 100%; 300 min, 90%; 270 min, 60%	(Booshehri et al., 2017)

### Summary

Currently, only very limited researchers have explored the pilot‐scale application of visible light photocatalysis. Although there are many visible light photocatalysts that have been developed and tested in the laboratory, the majority of the pilot‐scale test still focused on TiO_2_ and its composites.

The most common reactor used in pilot‐scale tests is CPC. It is a non‐imaging concentrator for solar energy collection (Kalogirou, 2014). Some studies use very simple reactors without proper design; some of them even put the water directly into a giant water vat. These reactors, including CPC, need further optimizations for real‐world visible light photocatalysis treatments: (1) the attachment of photocatalyst; (2) the mixture and separation of photocatalyst; (3) energy consumption of the reactor; and (4) durability.

## CONCLUSION AND PERSPECTIVES

Visible light active photocatalyst for water treatment has been widely examined due to its unique advantages. In this review, we have reviewed the current state of the art with a focus on materials, application, and pilot‐scale investigations. In addition, we have outlined the mechanisms of photocatalysis and reviewed a variety of synthesis, doping, and modification methods.

Significant progress in visible light active photocatalyst has been made during the past number of years. Many composite materials have been designed as well as the development of some new facile synthesis methods. Many studies have proved this technology as a promising advanced oxidation process (AOP) that has extensive application in urban, industrial, agricultural, and pharmaceutical wastewater treatment as well as natural water and drinking water treatment with potential for future further research. Visible light active photocatalyst can remove persistent organic pollutants, in addition to metallic ion reduction and microorganism degradation. Concurrent to water treatment applications, the visible light active photocatalyst is also used in air purification (J. U. Choi et al., 2019; Pichat, 2019), hydrogen production (Lee et al., 2019; Reddy et al., 2020), and chlorine production (Chehade et al., 2020). Notwithstanding, visible light active photocatalyst still faces many challenges that have to be addressed in future and are summarized below:

1. There is a lack of theoretical research on the photocatalyst modification mechanism. It is common knowledge that some widely used photocatalysts (i.e., TiO_2_) are not suitable for visible light active photocatalysis. Researchers have tried to modify it by shifting the absorption spectrum, reducing band gap energy, and slowing down the speed of charge recombination. Common methods include optimizing the photocatalyst's structure, doping other materials, and using support materials. However, the lack of a theoretical guide leads to unorganized modification studies, with some of them even having reported modified photocatalysts to be less effective than previous.
2. Research in photocatalysis‐enhanced solar disinfection is relatively limited despite millions of people having to use untreated drinking water in parts of the world with extensive solar exposure, and most solar disinfection studies did not apply photocatalyst. The majority of research in photocatalysis enhanced solar disinfection is only applied to common TiO_2_ and ZnO photocatalyst composite materials and very few of these have progressed to the pilot‐scale phase.
3. Many new visible light photocatalysts have been developed over the last few decades, but few have made it to the pilot‐scale application stage. Following laboratory and bench‐scale proof of concept, ability, and function, pilot‐scale experimental trials should be carried out before eventual application. Only a small number of pilot‐scale experimental trials have been documented in the literature with the majority using commercial TiO_2_ or other common composites. More pilot‐scale experimental trials are needed to expand the currently laboratory‐scale results for the full‐scale application of this promising sustainable technology.

## Data Availability

The data that support the findings of this study are available on request from the corresponding author. The data are not publicly available due to privacy or ethical restrictions.
